# APOL1 risk variants induce metabolic reprogramming of podocytes in patient-derived kidney organoids

**DOI:** 10.1016/j.stemcr.2025.102650

**Published:** 2025-10-02

**Authors:** Heein Song, Sébastien J. Dumas, Gangqi Wang, Lijun Ma, Franca Witjas, M. Cristina Avramut, Cathelijne W. van den Berg, Michael V. Rocco, Barry I. Freedman, Ton J. Rabelink, H. Siebe Spijker

**Affiliations:** 1 Department of Internal Medicine (Nephrology), Leiden University Medical Center, Albinusdreef 2, 2333ZA Leiden, the Netherlands; 2 Department of Internal Medicine, Section on Nephrology, Wake Forest University School of Medicine, Winston-Salem, NC, USA; 3 The Novo Nordisk Foundation Center for Stem Cell Medicine (reNEW), Leiden University Medical Center, Leiden, the Netherlands

**Keywords:** APOL1, kidney organoids, stem cells, mitochondria, podocyte

## Abstract

Carriers of two apolipoprotein L1 gene risk variants (RVs), termed G1 and G2, are at increased risk for chronic kidney disease. This study utilized induced pluripotent stem cells (iPSCs) derived from two patients homozygous for G1 and G2 to model human apolipoprotein L1 (APOL1)-mediated kidney disease (AMKD) in kidney organoids. Single-cell transcriptomic analysis and immunofluorescence imaging showed *APOL1* upregulation in podocytes after interferon-gamma (IFN-γ) treatment. Transcriptomics and spatial dynamic metabolomics demonstrated a significant reduction in oxidative phosphorylation and tricarboxylic acid (TCA) cycle activity, along with upregulation of glycolysis and hypoxia signaling in RV podocytes. Isolated RV glomeruli exhibited no increase in maximal respiration rate following IFN-γ treatment, while iPSC-derived RV podocytes displayed a reduced number of mitochondrial branches and shorter branch length. This model presents early metabolic reprogramming of RV podocytes upon inflammatory injury and compelling evidence that mitochondrial dysfunction plays a pivotal role in the early pathophysiology of AMKD.

## Highlights

• Established APOL1-mediated kidney disease model introducing both G1 and G2 patient iPSC-derived organoids
• APOL1 RV expression primarily impacts podocyte populations
• Multiomics analysis demonstrates metabolic reprogramming in RV podocytes
• RV podocytes display functional and structural mitochondrial impairment

## Teaser

Spijker and colleagues established a human model of APOL1-mediated kidney disease (AMKD) using kidney organoids generated from patient-derived iPSCs. Multiomics approach was applied to elucidate the pathogenic effects of APOL1 risk variants. The results demonstrate that APOL1-associated risk alleles primarily cause metabolic reprogramming of podocytes, characterized by structural and functional mitochondrial impairment.

## Introduction

The incidence rate of end-stage kidney disease (ESKD) is disproportionately high in African Americans. Their susceptibility is largely explained by genetic variation in the gene encoding apolipoprotein L1 (*APOL1*) (Genovese et al., 2010; Parsa et al., 2013). APOL1 protein functions as a trypanolytic factor, but *T. brucei rhodesiense* and *T. brucei gambiense* avoid lysis by producing the serum resistance-associated (SRA) protein (Cuypers et al., 2016; Itoku et al., 2024). APOL1 allelic variants alter the SRA-interacting domain, enabling evasion of SRA binding and restoration of host defense, which contributes to their high prevalence in individuals of West African descent (Cuypers et al., 2016; Itoku et al., 2024; Thomson et al., 2014). Two well-described variants are referred to as *APOL1* G1 (two missense variations p.S342G and p.I384M in almost complete linkage disequilibrium) and G2 (in-frame deletion of p.N388 and p.Y389), while G0 is considered wild type (Friedman and Pollak, 2011). Recently, it has been discovered that the risk of ESKD is markedly increased in the presence of two risk variant (RV) copies (G1G1, G2G2, or G1G2). Furthermore, a second hit (or modifier) such as HIV or COVID infection or systemic lupus erythematosus often precedes the development of *APOL1*-mediated kidney disease (AMKD) (Chen et al., 2017; Kopp et al., 2011; Larsen et al., 2021).

The clinical phenotype of AMKD is dominated by glomerular (podocyte) injury often resulting in focal segmental glomerulosclerosis or collapsing glomerulopathy (Freedman et al., 2021). APOL1 is present as a component of the circulating protein complex trypanosome lytic factors, as well as expressed intracellularly in the kidney, mainly in podocytes (Scales et al., 2020; Vanhamme et al., 2003; Weckerle et al., 2016). Interestingly, transplantation of *APOL1* high-risk genotype donor kidneys (irrespective of recipient genotype) yields significantly shorter allograft survival (Freedman et al., 2015; Reeves-Daniel et al., 2011). This suggests a central pathophysiological role for APOL1 expressed in the kidney. Although the disease course has become clinically defined in the last decade, the underlying disease-causing molecular mechanisms remain unclear. The absence of APOL1 in non-primates and its restricted presence only in a few higher primate species complicate the development of experimental models for studying APOL1-associated pathophysiology. Therefore, existing studies either assessed genetic overexpression of *APOL1* in HEK293T cells (O'Toole et al., 2018) and immortalized podocytes (Granado et al., 2017) or created transgenic mouse models (Beckerman et al., 2017; Bruggeman et al., 2016; Ryu et al., 2019). Most of these studies confirmed the pathogenic role of *APOL1*; however, elucidation of the main underlying mechanism has been debated with pathways that include inflammasome activation, lysosomal dysfunction, endoplasmic reticulum stress, mitochondrial dysfunction, Golgi trafficking, and forms of cell death (Daneshpajouhnejad et al., 2022; Freedman et al., 2021). Recently, approaches using human induced pluripotent stem cell (hiPSC)-derived organoid models have been utilized to study the potential molecular pathways that can be targeted for future therapeutics (Chun et al., 2022; Juliar et al., 2024; Liu et al., 2020; Nystrom et al., 2022).

In this study, we present the first *APOL1*-mediated kidney disease (AMKD) model incorporating patient-derived induced pluripotent stem cells (iPSCs) with both homozygous G1G1 and G2G2 risk genotypes. Gene correction was performed to generate a G0G0 control using CRISPR-Cas9, which provides the opportunity to study disease in a context isogenic to the patient. Integrated single-cell transcriptomic and metabolomic analysis revealed that *APOL1* expression induced by interferon-gamma (IFN-γ) results in mitochondrial respiration impairment and increased glycolysis in RV podocytes. Furthermore, we demonstrate that increased mitochondrial fragmentation is associated with the presence of APOL1 RV expression in iPSC-derived podocytes.

## Results

### Generation of *APOL1* RV patient iPSCs and the G0 control

**Figure 1:**
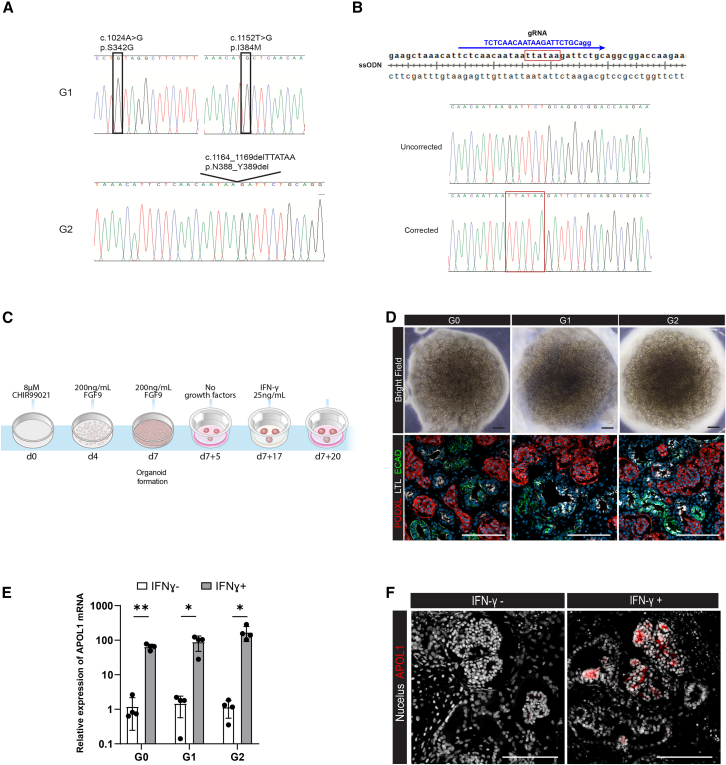
Establishment of AMKD patient and isogenic G0G0 iPSC lines to generate kidney organoids (A) Sequencing analysis of *APOL1* G1G1 and G2G2 patient-derived iPSCs. (B) Strategy for generating isogenic G0G0 control of G2 variant using CRISPR-Cas9 gene editing. Red outlined box represents the deletion site of the mutation. The sequence of gRNA is shown with the PAM sequence. Sequencing analysis shows correction of the 6 nucleotide deletion. ssODN, single-stranded oligodeoxynucleotide. (C) Kidney organoid differentiation and IFN-γ treatment scheme. Organoids were harvested for analysis 3 days after single-dose IFN-γ treatment. (D) Kidney organoids derived from G1, G2, and isogenic control G0 iPSCs. Upper: displays bright-field image of kidney organoids (scale bars, 500 μm). Lower: shows immunostaining images for PODXL (podocyte), LTL (proximal tubule), and ECAD (distal tubule). Scale bars, 100 μm. (E) RT-qPCR analysis of *APOL1* mRNA expression with or without IFN-γ treatment. Data are presented as mean ± SD (3 organoids per condition from 4 independent differentiations). ^∗^*p* < 0.05, ^∗∗^*p* < 0.01, by Student’s t test. (F) Immunostaining images display APOL1 protein expression after IFN-γ treatment. Scale bars, 100 μm.

Fibroblast cultures from skin biopsies in two female patients with AMKD were used to generate iPSCs: one with an *APOL1* G1G1 genotype and the other a G2G2 genotype. The G1G1 patient had stage 4 chronic kidney disease (CKD) with nephrotic syndrome and biopsy-proven collapsing glomerulopathy. The G2G2 patient had stage 5 CKD with microalbuminuria and kidney disease clinically attributed to hypertension. DNA from peripheral blood was genotyped on the Sequenom platform for the two SNPs in the G1 locus (rs73885319 and rs60910145) and the indel platform for the G2 locus (rs71785313) using custom arrays designed at Wake Forest (Langefeld et al., 2018). G1 and G2 cells were visually inspected for quality control. Following successful reprogramming into iPSCs, *APOL1* RV sequences were confirmed by Sanger sequencing in two G1 and two G2 lines (Figure 1A). To generate G0 control iPSCs, the G2 line was genetically corrected using CRISPR-Cas9 ribonucleoprotein and single-stranded DNA-mediated homologous recombination containing the wild-type sequence (Figure 1B). Flow cytometry was used to confirm the expression of pluripotency markers, and immunofluorescence staining was used to verify successful differentiation into ectoderm, mesoderm, and endoderm in all iPSC lines (Figure S1A). Furthermore, all iPSC lines exhibited normal karyotype and morphological appearance (Figures S1B and S1C).

### Patient-derived iPSCs differentiate into kidney organoids that express *APOL1* after IFN-γ

iPSCs were differentiated into kidney organoids according to a previously published protocol (Figures 1C and 1D) (van den Berg et al., 2018). Organoids from all three lines were morphologically similar and contained both proximal and distal segments as confirmed by immunoreactivity for podocytes (PODXL) and proximal and distal tubular markers (LTL and ECAD, respectively) (Figure 1D). *APOL1* gene and APOL1 protein expression levels were scarcely detected under regular culture conditions. To induce *APOL1* expression, a single dose of 25 ng/mL IFN-γ, an established risk factor for AMKD, was added at day 7+17 (Figure 1C). Quantitative PCR showed induction of *APOL1* expression in G0 and RV organoids (Figure 1E). Protein expression of APOL1 was confirmed after IFN-γ exposure (Figure 1F). Kidney organoids at day 7+20 were used to characterize the early pathological effect of *APOL1* RV induction.

### Podocytes are the principal cell type impacted by APOL1 RV expression

**Figure 2:**
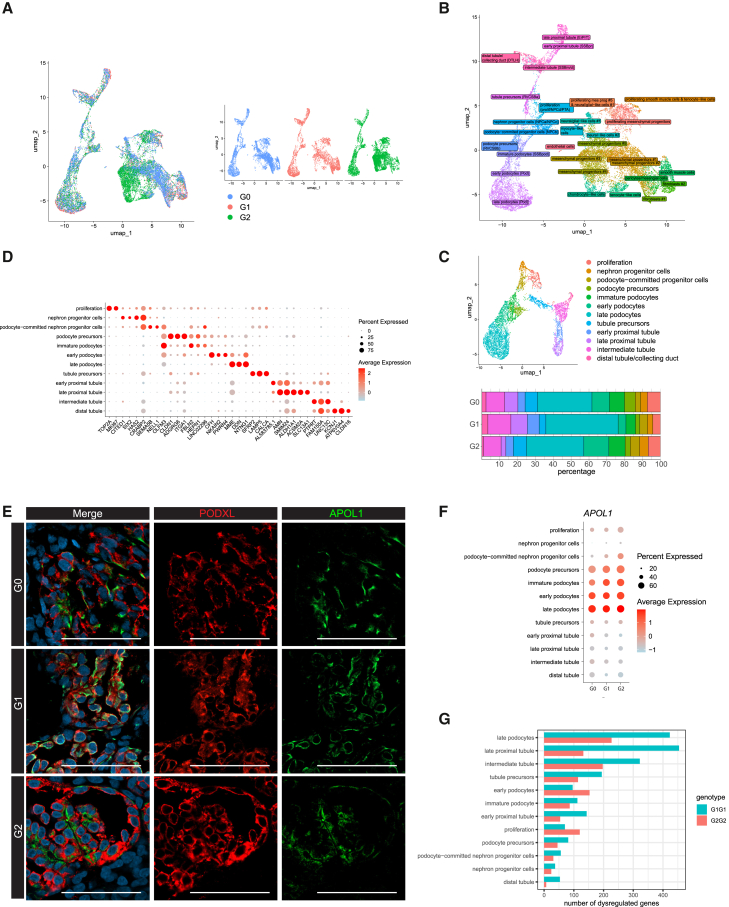
*APOL1* is primarily expressed in podocyte populations, and RV podocytes display the highest number of dysregulated genes (A) UMAP visualization of all cells obtained from G0, G1, and G2 iPSC-derived kidney organoids at day 7+20. Color-coded by iPSC line. (B) UMAP visualization of 30 cell clusters, color-coded by cell population. (C) Dot plot showing marker gene expression in nephron cell clusters. (D) Upper: shows UMAP visualization of nephron cells from kidney organoids, color-coded by the main cell population. Lower: shows relative cluster quantification for nephron cells of kidney organoid from each iPSC line, showing similar proportions of nephron cell populations between RV and G0 organoids. (E) Immunostaining shows that *APOL1* expression is mainly co-localized with PODXL^+^ (podocyte) cells. Scale bars, 50 μm. (F) Dot plot presenting *APOL1* expression in nephron cell clusters. *APOL1* expression was observed mainly in podocyte populations (podocyte precursor, immature podocyte, early podocyte, and late podocyte) regardless of the *APOL1* genotype. (G) The number of dysregulated genes per cell cluster. Genes were considered dysregulated when adjusted *p* value < 0.05, and log2FC > 0.2 or log2FC < −0.2.

To better understand the expression patterns of *APOL1* in kidney organoids, single-cell RNA sequencing (scRNA-seq) was performed on IFN-γ-treated organoids harvested at day 7+20. All three cell lines were cultured in parallel for this analysis. Single-cell transcriptome data of 20,174 high-quality organoid cells were analyzed (wild-type G0: 6,572 cells with 5,559 detected genes/cell on average; RV G1: 5,185 cells with 5,671 detected genes/cell on average; RV G2: 8,417 cells with 5,391 detected genes/cell on average). Unsupervised clustering revealed 30 populations characterized by unique transcriptome profiles corresponding to different cell types and states, including nephron cells, mesenchymal cells, and endothelial cells (Figures 2A–2C and S2A). More specifically, lineage-specific marker expression identified 12 distinct nephron cell populations including 4 podocyte clusters at different developmental stages of maturation (podocyte precursors, immature podocytes, early podocytes, and late podocytes), 5 tubule clusters (early and late proximal tubule, intermediate tubule and distal tubule, and tubule precursors) and nephron progenitors, and 17 mesenchymal populations including progenitors, fibroblasts, smooth muscle cells, pericytes and endothelial cells, small off-target neural cells, chondrocytes, tenocytes, and myocytes (Figure 2B). Identical cell populations were observed in both RV and G0 organoids, although their relative abundances varied; a higher proportion of fibroblasts were present in G1 RV organoids and of mesenchymal progenitor populations in G2 RV organoids (Figures 2A–2D, S2B, and S2C). *APOL1* was abundantly expressed in the podocyte clusters during all developmental stages and to a lesser extent in tubule cells, smooth muscle cells, and endothelial cells (Figure S2D). Podocyte APOL1 expression was confirmed at the protein level via co-immunoreactivity of APOL1 and PODXL (Figures 2E and S2E). Among podocytes, *APOL1* expression was highest in the most mature late podocytes (Figure 2F), and late podocyte clusters showed the highest number of dysregulated genes among all nephron cell populations in RVs (Figure 2G).

After pooling data from both G1 and G2, differential gene expression analysis of RV compared to G0 organoid cells was performed, correcting for cell-line differences.

### Differential gene expression analysis reveals energetic dysregulation in *APOL1* RV podocytes

**Figure 3:**
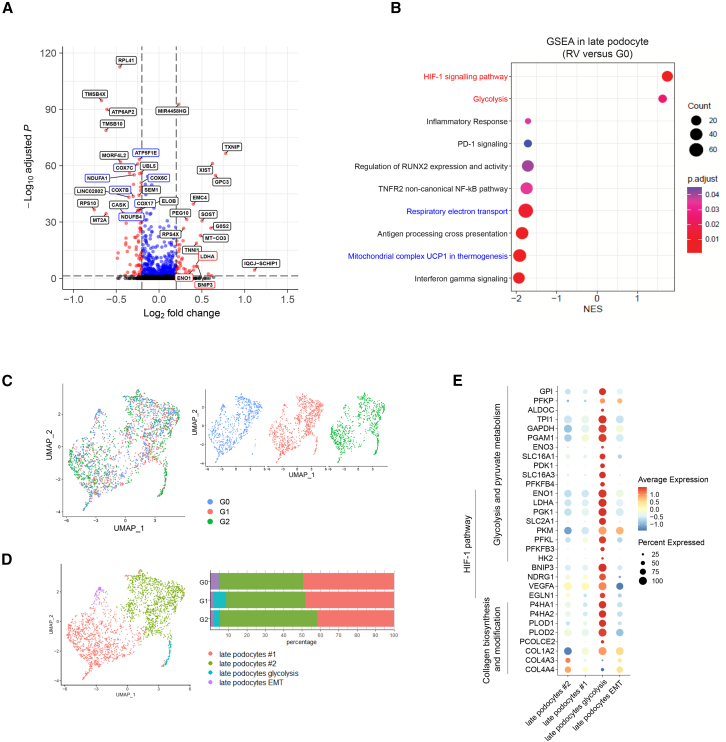
*APOL1* RV expression significantly alters metabolism-related gene sets in organoid podocytes (A) Differential gene expression analysis (DGEA) in late podocyte populations of kidney organoids. G1 and G2 organoid cells were pooled as RV cells and compared to G0 organoid cells. Blue box indicates nuclear OXPHOS genes and red box marks glycolysis and hypoxia-inducible factor (HIF)-1 signaling pathway genes. (B) Gene set enrichment analysis (GSEA) for RV late podocytes versus G0 late podocytes. NES, normalized enrichment score. (C) UMAP visualization of late podocyte subclustering. Color coded by cluster (left) and by *APOL1* variant type (right). (D) Relative cluster quantification of late podocyte subclusters, showing late podocyte glycolysis cluster is nearly absent in G0. (E) Dot plot representing marker gene expression in late podocyte subclusters, characterized by glycolysis, pyruvate metabolism genes, HIF-1 signaling pathway genes, and collagen biosynthesis and modification genes.

Because late podocytes showed the highest *APOL1* expression and were the most affected mature cell type in *APOL1* RVs, we further explored the differentially expressed genes between RV and G0 late podocytes. A total of 45 upregulated and 72 downregulated genes were identified in RV compared to G0 (Table S1). Among the most differentially expressed genes, *MIR4458HG*, *TXNIP*, *GPC3*, *EMC4*, *PEG10*, *BNIP3*, *ENO1*, and *LDHA* showed significantly higher expression in RV late podocytes. In contrast, genes encoding a subunit of the mitochondrial ATP synthase (*ATP5MD*, *ATP5MG*, *ATP5MF*, and *ATP5F1E*), cytochrome-*c*-oxidase (*COX7A2*, *COX7C*, *COX6C*, *COX7B*, and *COX17*), and mitochondrial complex I or NADH dehydrogenase (ubiquinone) subunits (*NDUFA1*, *NDUFB2*, and *NDUFB4*) showed lower expression in RV compared to G0 late podocytes (Figure 3A). Next, gene set enrichment analyses comparing RV and G0 late podocytes revealed downregulated response to interferon (IFN-γ signaling, antigen cross-presentation, and inflammatory response), suggesting that *APOL1* RV affects the pro-inflammatory response to IFN-γ in podocytes. Further, gene sets for oxidative phosphorylation (OXPHOS) and mitochondrial respiration were significantly downregulated, while hypoxia signaling and glycolysis were upregulated (Figure 3B), indicating a metabolic switch from OXPHOS to glycolysis in IFN-γ-stimulated RV late podocytes. Separate comparison of G1 vs. G0 and G2 vs. G0 confirmed that commonly downregulated gene sets in both G1 and G2 were related to mitochondrial respiration, indicating that G1 and G2 RVs share similar pathophysiology (Figures S3A and S3B).

Further in-depth subclustering analysis of the late podocyte populations revealed 4 distinct subclusters (late podocytes #1 and #2, late podocytes undergoing endothelial-to-mesenchymal transition, and late podocyte glycolysis) (Figures 3C and 3D). Interestingly, the late podocyte glycolysis (small tail-like) cluster was identified in both G1 and G2 but was nearly absent in G0 late podocytes (Figure 3D), indicating an RV-specific podocyte phenotype. This unique subcluster was characterized by a strong glycolytic and hypoxic signature, expressing genes such as *SLC2A1*, *ENO1*, *LDHA*, and *BNIP3* (Figure 3E) and showed elevated expression of gene signatures associated with collagen biosynthesis and modification, such as *P4HA1*, *P4HA2*, *PLOD1*, *PLOD2*, and *COL1A2*. To translate kidney organoid transcriptome findings to human glomerular transcriptome data from kidney biopsy, the publicly available dataset from the NEPTUNE cohort was analyzed for glycolytic genes (McNulty et al., 2022). Genome-wide *APOL1* correlation analysis in this clinical dataset showed that the genes encoding glycolytic enzymes had strong positive correlation with the expression level of *APOL1* RV not with wild-type *APOL1* (Figure S3C; Table S2). In summary, *APOL1* RV organoids displayed lesser response to IFN-γ and an altered metabolic transcriptome characterized by downregulated OXPHOS and upregulated glycolysis pathways in RV podocytes. The transcriptional characteristics of increased glycolysis in RV podocytes resembled those in kidney biopsy data in AMKD.

### Lipidomic profiling spatially identifies mature podocyte populations

**Figure 4:**
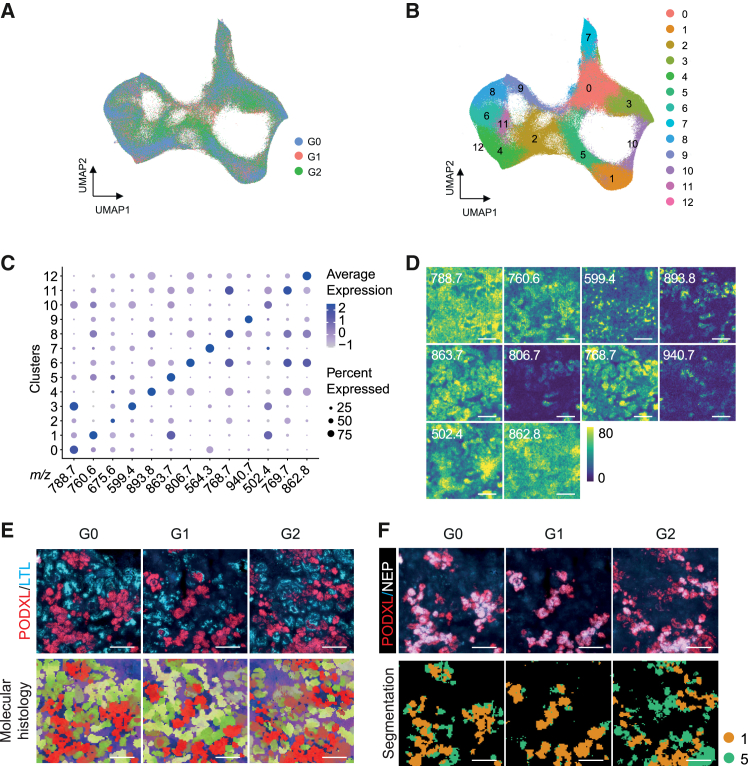
Spatial identification of mature podocytes by specific lipid profiles (A) UMAP visualization of integrated lipidomics data of day 7+20 kidney organoids from G0, G1, and G2 iPSCs. Three organoids from 3 independent differentiations for each cell line were analyzed, showing similar clustering between 3 genotypes. (B) 13 unique clusters were identified by lipid profile. (C) Dot plot representing lipid expression of cluster-enriched signatures. (D) Representative images of distribution of lipid species in kidney organoids, recorded by MALDI-MSI (5 × 5 μm^2^ pixel size). Scale bars, 200 μm. (E) Post-MALDI-MSI immunostaining (upper) and representative molecular histology of kidney organoids, generated from 3D UMAP analysis on the basis of lipid profiles. Each pixel is assigned with RGB colors based on its location in 3D UMAP. Scale bars, 200 μm. (F) Post-MALDI-MSI staining of PODXL and Neprilysin (NEP) (upper) and representative spatial segmentation image (lower) showing spatial lipid heterogeneity between PODXL^+^/NEP^+^ mature podocytes and PODXL^+^/NEP^−^ immature podocytes. Spatial segmentation colors match the color scheme of the UMAP in (B). Scale bars, 200 μm.

To further assess the changes in cell-type-specific metabolism, matrix-assisted laser desorption/ionization mass spectrometry imaging (MALDI-MSI) combined with isotope tracing was performed, as previously reported (Wang et al., 2022; Wang et al., 2022). The metabolite and lipid profiles were assessed in a spatial context on fresh frozen organoid sections. First, we set out to spatially identify podocyte populations. Dimensionality reduction of the lipidome was performed by applying a uniform manifold approximation and projection (UMAP) that revealed highly overlapping clusters of the three *APOL1* genotypes (Figures 4A and S4A). Next, 13 unique independent cell clusters were identified based on the lipid profiles (Figures 4B and 4C) and were spatially mapped back to the organoid section (Figure 4D). After performing MSI measurements, sections were immunostained for PODXL and LTL to distinguish podocyte and proximal tubule segments. Integration of immunostaining and the spatial lipidomic profiles created a uniform overlap of molecular histology with PODXL- or LTL-immunoreactive cells, comparable throughout the three genotypes (Figure 4E). Next, further discrimination of immature and mature podocyte populations was performed. In transcriptome analysis, late podocytes strongly expressed both *PODXL* and the endopeptidase *MME* (encoding Neprilysin [NEP] gene) (Figure S4B). Subsequent immunostaining identified a subset of PODXL and NEP double-positive podocytes (PODXL^+^/NEP^+^), which were regarded as mature podocytes. The combined immunostaining and lipidomic results indicated that lipid cluster 1, characterized by m/z 760.6, overlapped with PODXL^+^/NEP^+^ mature podocytes (Figure 4F). Meanwhile, lipid cluster 5 correlated with PODXL^+^/NEP^−^ cells, therefore considered to be immature podocytes (Figure 4F). These results show that the lipidomic profile can spatially identify podocytes at different stages of development in kidney organoids.

### Metabolic analysis indicates increased glycolysis with lower TCA cycle activity and reduced respiration in *APOL1* RV podocytes

**Figure 5:**
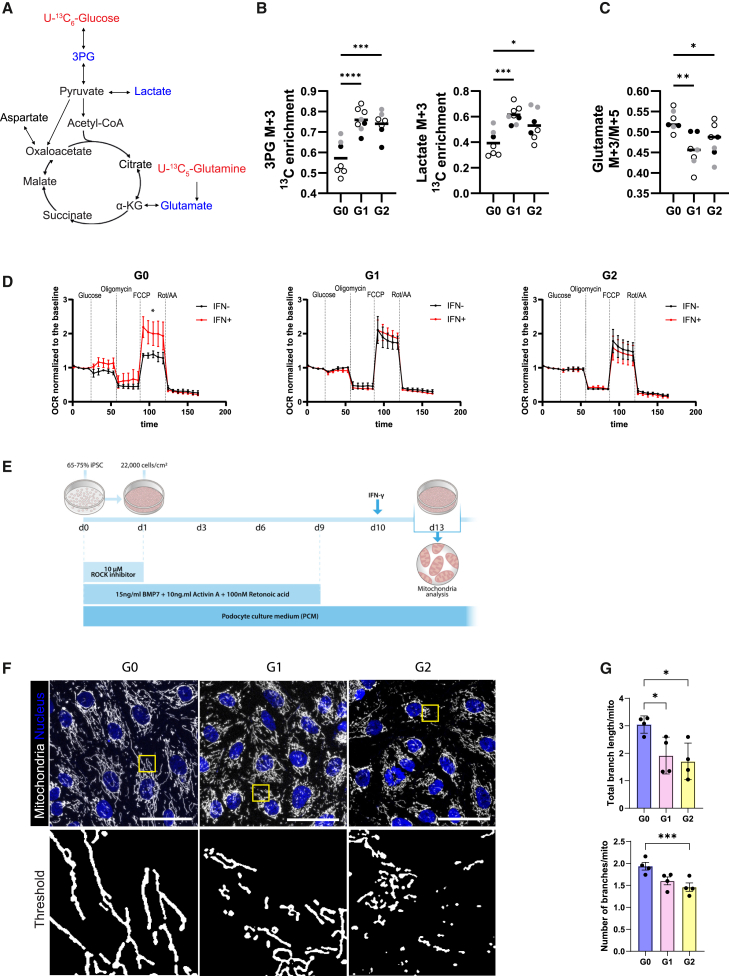
RV mature podocytes display higher glycolytic activity, lower TCA cycle activity, and mitochondrial impairment (A) The schematic representation of spatial dynamic metabolic measurements using U-^13^C_6_-glucose and U-^13^C_6_-glutamine on mature podocytes in kidney organoids (labeled glucose and glutamine depicted in red, measured metabolites in blue). (B) ^13^C_6_-glucose enrichment in 3PG and lactate. One-way analysis of variance (ANOVA) with Dunnett’s multiple comparison test was performed. 3PG, 3-phosphoglycerate. ^∗^*p* < 0.05, ^∗∗∗^*p* < 0.001, ^∗∗∗∗^*p* < 0.0001. (C) Ratio of glutamate M+3 and glutamate M+5. One-way ANOVA with Dunnett’s multiple comparison test was performed. ^∗^*p* < 0.05, ^∗∗^*p* < 0.01. (D) Mitochondrial stress test in G0, G1, and G2 glomeruli with and without IFN-γ induction. Oxygen consumption rate (OCR) was measured using Seahorse XF-96 extracellular flux analyzer in the presence of glucose, oligomycin, FCCP, and Rot/AA and normalized to basal OCR. Data are expressed as mean ± SEM (3 independent differentiations). The area under the curve (AUC) following FCCP treatment was calculated, and a paired t test was performed to compare IFN-γ-treated and untreated glomeruli isolated from kidney organoids, ^∗^*p* < 0.05. (E) Podocyte differentiation from iPSC scheme. IFN-γ was added at day 10, and mitochondria were analyzed on day 13. (F) Representative image of mitochondrial morphology from G0, G1, and G2 podocytes. Original image (upper) and images upon thresholding (lower) are shown. Scale bars, 50 μm. (G) Mitochondrial morphology was quantified as number of branches and total branch length per mitochondria. One-way ANOVA with Dunnett’s multiple comparison test was performed. ^∗^*p* < 0.05, ^∗∗∗^*p* < 0.001.

Based on the spatial identification following co-registration of immunostaining and lipid clustering, we next focused on changes in glycolysis and the tricarboxylic acid (TCA) cycle in the mature podocyte population. To dynamically assess the metabolite measurements, the labeled nutrients [U-^13^C]glucose and [U-^13^C]glutamine were added in separate wells to RV and G0 organoids for 4 h before snap-freezing the samples (Figure 5A). First, [U-^13^C]glucose was used to assess its contribution to glycolysis and the TCA cycle. Significantly higher ^13^C enrichment was present in 3-phosphoglycerate (M+3) and lactate (M+3) derived from glucose labeling in RV mature podocytes compared to G0, implicating a higher glycolytic flux in RV podocytes (Figure 5B). Next, to assess the dynamics of the TCA cycle, G0 and RV organoids were incubated with [U-^13^C]glutamine. As the conversion from glutamine M+5 to glutamine M+3 goes through one oxidative TCA cycle, the ratio of glutamate M+3 and glutamate M+5 was used to assess TCA cycle activity. Both G1 and G2 showed lower M+3/M+5 ratio, indicating overall decreased TCA cycle activity compared to G0 (Figure 5C).

Together, the spatial metabolomics analysis corresponded to the increased glycolysis observed in the transcriptome analysis for both G1 and G2 RV, while glutamine labeling showed reduced TCA cycle in G1 and G2 RV podocytes compared to G0.

### Expression of *APOL1* RVs impairs mitochondrial respiration exhibited with fragmented mitochondria

As transcriptomic analyses suggested that *APOL1* RV expression in podocytes leads to reduced OXPHOS, the mitochondrial respiration rate in RV and G0 podocytes was compared using the Agilent Seahorse XF assay. Since tubule cells are highly metabolically active and would likely conceal changes in podocyte respiration, podocytes were first isolated by dissociating the organoids and subsequently isolating glomeruli at day 7+20, as described previously (Hale et al., 2018). Glomeruli were isolated as intact cell clusters highly enriched for podocytes (Figure S4C). In G0 isolated glomeruli, the maximal respiration rate significantly increased upon IFN-γ treatment (Figure 5D). However, increased mitochondrial respiration after IFN-γ was blunted in both G1 and G2 glomeruli indicating impaired response of the electron transport chain in the context of *APOL1* RV (Figure 5D). Mitochondria undergo dynamic morphological changes through a process of fission and fusion to maintain optimal mitochondrial function. Therefore, we hypothesized that APOL1 RV expression would impair control of mitochondrial dynamics. Due to the limitations of 3D organoids for analyzing mitochondria morphology, we differentiated 2D podocytes from hiPSCs according to a previously published protocol (Murphy et al., 2024). At the end of the differentiation, hiPSC-derived podocytes showed larger cell body and expression of podocyte markers synaptopodin and PODXL (Figures S5A and S5B). As in the organoid, *APOL1* was highly expressed when 2D iPSC-derived podocytes were incubated with IFN-γ and mitochondrial morphology was analyzed 3 days after the induction (Figures 5E and S5C). Interestingly, a significant reduction in the mitochondrial branch length and the number of branches was observed with APOL1 RV iPSC-derived podocytes compared to G0, indicating that mitochondria were more fragmented in RV podocytes (Figures 5F, 5G, and S5D). In sum, these data indicate that mitochondria are more fragmented after induction of *APOL1* RV, and this appears to impair the increased demand for mitochondrial respiration.

## Discussion

The mechanism by which *APOL1* RVs contribute to kidney disease remains a subject of active investigation. Progress in elucidating these mechanisms has been challenging due to the absence of an optimal model, as *APOL1* is exclusively expressed in humans and nonhuman primates. In this study, a disease model of AMKD was developed using patient-derived iPSCs with a G1G1 and G2G2 genetic background. Confounding genetic factors were controlled by generating control G0 iPSCs isogenic to G2 using CRISPR-Cas9 gene editing. We detected metabolic dysfunction in *APOL1* RV podocytes after IFN-γ treatment; glycolytic flux was increased at the expense of mitochondrial respiration, and mitochondria fragmentation was observed.

Integration of single-cell level multi-omics on human kidney organoid and iPSC-derived podocyte models recapitulated human kidney disease. This provided the opportunity to elucidate the potential mechanism of AMKD pathophysiology, typically regarded as a podocytopathy. This study shows increased expression of *APOL1* in podocytes following exposure to IFN-γ, both at the transcript and protein levels, and the highest number of differentially expressed genes were found in podocytes. Unbiased analysis of single-cell transcriptomics 3 days after IFN-γ treatment was performed to assess early pathological changes; metabolic dysregulation and hypoxia signaling were observed. These findings were in line with the transcriptomic analysis of dissected glomeruli from AMKD patient biopsies in NEPTUNE (McNulty et al., 2022), as APOL1 RV showed positive correlation with glycolysis and weaker co-expression with OXPHOS and the electron transport chain. Detailed assessment of cellular metabolism is hindered by its dynamic change over time and metabolic heterogeneity between cell types. To overcome this, spatial metabolic information and dynamic isotope tracing at single-cell resolution were combined. Importantly, even though the G0 control was isogenic to G2, differences in glucose enrichment were also observed in G1 podocytes, confirming the strong signature of *APOL1* RV on podocyte pathology largely irrespective of the genetic background.

RV and G0 organoids had similar cell compositions, but G1 and G2 RV organoids showed increased fibroblast and mesenchymal progenitors. A unique RV podocyte subpopulation also displayed elevated glycolytic, hypoxic, and extracellular matrix remodeling gene expression. While the enrichment of these cell types in RV organoids may be a reflection of glomerulosclerosis observed in AMKD, further research is needed to address this observation (Larsen et al., 2015).

Previous studies in mice have shown that podocytes mainly depend on anaerobic glycolysis under physiological conditions (Brinkkoetter et al., 2019). We assessed mitochondrial respiration in glomeruli isolated from organoids and showed that the oxygen consumption rate (OCR) increased in G0 organoids upon IFN-γ exposure. This suggests that while podocytes are normally dependent on glycolysis, the stress response implicates increased energy demand using OXPHOS, which appears impaired in the context of *APOL1* RV. Interestingly, monocytes display a similar response to IFN-γ by increasing respiration rate through reprogramming of NAD+ metabolism (McCann et al., 2022).

The complex three-dimensional structure of organoids makes mitochondrial imaging and assays challenging. Therefore, we differentiated podocytes from the hiPSCs (patient-derived G1 and G2 and isogenic G0) to investigate the effect of *APOL1* RV on mitochondrial dynamics. Quantification of mitochondrial staining revealed reduced mitochondrial branch numbers and branch length in both *APOL1* G1 and G2 RVs compared to the isogenic control, indicating increased mitochondrial fragmentation. Mitochondrial fusion and fission dynamics help maintain the optimal OXPHOS activity; therefore, balancing this process is crucial for cell survival and optimally functioning mitochondria (Adebayo et al., 2021; Chan, 2020). Mitochondrial fusion enhances the mitochondrial oxidative capacity in response to stress or increased energy demand, enabling efficient ATP production and the dilution of damaged mitochondrial components. Mitochondrial fission facilitates the isolation and removal of severely damaged mitochondria, thereby maintaining mitochondrial network integrity. Previous reports suggested that, in the presence of *APOL1* G1 and G2 RVs, excessive mitochondrial fission occurs without adequate compensation via mitophagy, which can trigger cell death pathways (Archer, 2013; Youle and van der Bliek, 2012). Our data suggest that dysfunctional mitochondrial dynamics, induced by *APOL1* G1 and G2 RVs, contribute to the pathogenesis of AMKD.

There has been substantial investigation into the relationship between *APOL1* RVs and mitochondrial dysfunction. APOL1 protein has been found to localize to mitochondria (Granado et al., 2017; Shah et al., 2019). The induction of *APOL1* G1 and G2 expression results in a significant reduction in the maximal mitochondrial respiration rate and respiratory reserve capacity compared to cells expressing the *APOL1* G0 variant (Granado et al., 2017; Ma et al., 2017). *APOL1* RV overexpression in HEK293 cells increased mitochondrial fission by activation of DRP1 (Ma et al., 2020). Lastly, a recent discovery showed that *APOL1* can form large oligomers upon import into mitochondria, inducing mitochondrial permeability transition pore activation and ultimately cell death (Shah et al., 2019). While research increasingly highlights the contribution of mitochondrial dysfunction as a critical mechanism underlying the pathogenic effects of *APOL1* RVs, these models have generated debate on the mechanism of action of *APOL1* and the extent to which artificial (over)expression correlates to human pathophysiology (Beckerman et al., 2017; Bruggeman et al., 2016; Granado et al., 2017; Ma et al., 2017). Here, our data add strong evidence using a multi-omics approach that mitochondrial impairment is the key cellular disturbance in podocytes. It remains unclear whether *APOL1* RVs directly or indirectly increase mitochondrial fragmentation and whether they promote mitochondrial fission or impair fusion.

The relative immaturity of nephron cell types in organoids remains a limitation for disease modeling. Previous studies showed kidney organoids to be comparable to end of first trimester embryonic nephrons. However, a comparison between organoid-derived glomeruli and 2D cultured podocytes revealed that organoids are more comparable to a human kidney, in both apicobasal polarity and transcriptomic profiles (Hale et al., 2018). These findings indicate that patient-derived organoids offer the best platform for human disease modeling with currently available technologies. Previous reports suggest a pathogenic role for *APOL1* expressed in endothelial cells in sepsis or circulating immune cells, which this model could not investigate (Wu et al., 2021; Zhang et al., 2021). The organoids we generated had few endothelial and immune cells, providing insufficient power to reliably investigate these cell types. Future studies could use our patient-derived iPSCs to directly differentiate endothelial cells or adjust differentiation protocols to enhance the presence of endothelial cells in kidney organoids (Koning et al., 2020).

Overall, this study provides valuable insights that point toward mitochondrial impairment as a central driver of the metabolic reprogramming observed in *APOL1* RV podocytopathy and a key pathogenic event in AMKD, thereby paving the way for future therapeutic strategies.

## Methods

### Generation of iPSC lines

Skin fibroblasts were obtained at Wake Forest University School of Medicine from two patients with confirmed G1G1 or G2G2 genotypes, in accordance with the local Institutional Review Board (IRB00060510). Patient iPSC lines LUMC0214iAPOL (G1G1) and LUMC0216iAPOL (G2G2) were generated using Sendai virus reprogramming as reported (Nishimura et al., 2011). The G0 control iPSC lines were generated from LUMC0216iAPOL using electroporation of the Cas9-RNP complex and single-stranded oligodeoxynucleotide (ssODN) as HDR donor. The details of guide RNA used and the template for homologous recombination template (ssODN) are listed in Table S3.

Two corrected clones (ISO01LUMC0216iAPOL05 and ISO03LUMC0216iAPOL05) were identified using restriction enzyme digestion of Psil restriction site, confirmed by Sanger sequencing, and used as isogenic controls. As non-isogenic G0 controls, iPSC0028 (Sigma) and LUMC0072iCTRL01 (generated from LUMC hiPSC core facility) iPSCs were used. The information of the iPSC lines and the nomenclature used in the manuscript are provided in Table S4. The iPSCs are registered in hPSCreg, and the nomenclature follows a system adopted at LUMC, based on the publication (Luong et al., 2011).

### iPSC culture and organoid differentiation

Detailed iPSC culture and organoid differentiation procedures are provided in supplemental experimental procedures. To induce *APOL1* overexpression, a single dose 25 ng/mL IFN-γ (R&D Systems) was added to the medium on day 7+17.

### iPSC-derived podocyte differentiation

The iPSCs were differentiated into podocytes using previously reported protocol as described in supplemental methods (Murphy et al., 2019, 2023; Rauch et al., 2018).

### Immunofluorescence analysis

See supplemental methods.

### Mitochondrial morphology analysis

Mitochondrial morphology was analyzed using Fiji/ImageJ integrated with Mitochondria Analyzer plug-in (Chaudhry et al., 2020). Auto thresholding was applied to the images before analysis with the mitochondria analyzer. A minimum of 10 randomly selected cells per condition were analyzed from 4 independent differentiations.

### mRNA isolation and qPCR

See supplemental methods.

### scRNA-seq sample preparation

5 IFN-γ-treated kidney organoids from each cell line were harvested on day 7 + 20 and dissociated to single cells as reported (also see supplemental methods) (Koning et al., 2022). The single-cell suspensions obtained from dissociated kidney organoids were converted to barcoded scRNA-seq libraries using the Chromium Single Cell 3′ Library, Gel Bead & Multiplex kit and Chip kit (10× Genomics).

### scRNA-seq and data analysis

Libraries were sequenced on an Illumina NovaSeq6000 S4 flow cell using a 300 cycle kit and v.1.5 chemistry and aiming for at least 50k reads/cells. Paired-end sequencing (28 + 91+8 bp) was used to determine (1) the cell barcode and UMI, (2) the transcript, and (3) the sample index, respectively. Fastq files were demultiplexed using bcl2fastq v.2.2 and processed using Cellranger-7.0.0. Downstream analysis was performed with R (v.4.3.1) in RStudio for Windows (v.2023.06.1 build 524). Filtered data matrices of each sample were imported from the CellRanger output into R using the *Read10X()* function from the Seurat package (v.4.4.0). Detailed information of data analysis is provided in supplemental experimental procedures.

### Isolation of glomeruli from kidney organoids

See supplemental methods.

### OCR measurement

OCR was analyzed using a XFe 96 extracellular flux analyzer (Seahorse Bioscience). The detailed information is provided in supplemental experimental procedures.

### Spatial dynamic metabolomics sample preparation and matrix deposition

Kidney organoids were incubated in a well-defined glucose-free and glutamine-free DMEM medium (Gibco A1443001) supplemented with 2% FCS, 14 mM glucose, and 2 mM glutamine for 4 h at 37°C with 5% CO2. For the 13C-labeling incubation, identical amounts of either [U-^13^C]glucose or [U-^13^C]glutamine were used to replace similar un-labeled nutrients in each medium. After 4 h of incubation, kidney organoids were quenched with liquid N2 and stored at −80°C. At least 2 organoids were used for each differentiation, and 3 separate differentiations of kidney organoids were analyzed for each condition.

Cryopreserved tissue biopsies were embedded in 10% gelatin and cryosectioned into 10-μm-thick sections at −20°C. Sections were thaw-mounted onto indium-tin-oxide-coated glass slides (VisionTek Systems) and stored at −80°C. Detailed description of tissue preparation and matrix deposition is provided in supplemental experimental procedures.

### MALDI-MSI measurement and analysis

MALDI-TOF/TOF-MSI was performed using a RapifleX MALDI-TOF/TOF system (Bruker Daltonics). Negative-ion-mode mass spectra were acquired at a pixel size of 9 μm (*x*, *y*) using a beam scan area of 5 × 5 μm and a mass range of m/z 80–1,000. Detailed measurement procedure is provided in supplemental experimental procedures.

After the MALDI-MSI data acquisition, excess matrix was removed, and immunofluorescence staining was performed on the tissue slide as described in supplemental methods. The stained tissues were scanned using a digital slide scanner (3D Histech Pannoramic MIDI Scanner, Sysmex). Digital scanned images were aligned with the MALDI-MSI data. The detailed MSI data processing and analysis procedures are provided in supplemental experimental procedures.

## Resource availability

### Lead contact

Requests for further information and resources should be directed to and will be fulfilled by the lead contact, H. Siebe Spijker (h.s.spijker@lumc.nl).

### Materials availability

iPSC lines generated in this study can be made available on request.

### Data and code availability

• The data of single-cell transcriptomics supporting the findings of this study are available in European Genome-Phenome Archive at accession ID: Study EGAS50000001223, Dataset EGAD50000001743.
• The exported and processed MSI data for this study were deposited in FigShare at DOI: https://doi.org/10.6084/m9.figshare.26064208.

## Acknowledgments

The authors thank Christian Freund (hiPSC hotel, LUMC, Leiden, the Netherlands), Christiaan Arendzen (hiPSC hotel, LUMC, Leiden, the Netherlands), and the LUMC iPSC core facility for providing hiPSC lines. We acknowledge the support of Yun Suk Chae (LUMC, Leiden, the Netherlands), Anneloes Verwey (LUMC, Leiden, the Netherlands), Rianne van Nieuwland (LUMC, Leiden, the Netherlands), Annelies Boonzaier-van der Laan (LUMC, Leiden, the Netherlands), Manon Zuurmond (LUMC, Leiden, the Netherlands), 10.13039/501100002997Dutch Kidney Foundation (Kolff 20OK017), and Novo Nordisk Foundation Center for Stem Cell Medicine (reNEW, supported by the Novo Nordisk Foundation grant [NNF21CC0073729]).

## Author contributions

Conceptualization, H.S., S.J.D., L.M., F.W., C.W.v.d.B., M.V.R., B.I.F., T.J.R., and H.S.S.; methodology, H.S., S.J.D., G.W., and M.C.A.; investigation, H.S., S.J.D., G.W., and H.S.S.; visualization, H.S., S.J.D., and G.W.; supervision, T.J.R. and H.S.S.; writing – original draft, H.S. and H.S.S.; writing – review and editing, H.S., S.J.D., G.W., L.M., M.C.A., C.W.v.d.B., M.V.R., B.I.F., T.J.R., and H.S.S.

## Declaration of interests

Wake Forest University Health Sciences and B.I.F. have rights to a US patent related to *APOL1* gene testing (https://www.apol1genetest.com). B.I.F. is a consultant for and receives research support from AstraZeneca.

## Supplemental information

Document S1. Figures S1–S5, Tables S2–S5, and supplemental methods

Table S1. Significantly upregulated or downregulated genes in G1+G2 compared to G0, G1 vs. G0, and G2 vs. G0 Log2 Fold change(Log2FC) threshold set at 0.2 and −0.2. Adjusted p value < 0.05

Document S2. Article plus supplemental information

## References

[bib1] Adebayo M., Singh S., Singh A.P., Dasgupta S. (2021). Mitochondrial fusion and fission: The fine-tune balance for cellular homeostasis. Faseb j.

[bib2] Archer S.L. (2013). Mitochondrial dynamics--mitochondrial fission and fusion in human diseases. N. Engl. J. Med..

[bib3] Beckerman P., Bi-Karchin J., Park A.S.D., Qiu C., Dummer P.D., Soomro I., Boustany-Kari C.M., Pullen S.S., Miner J.H., Hu C.A.A. (2017). Transgenic expression of human APOL1 risk variants in podocytes induces kidney disease in mice. Nat. Med..

[bib4] Brinkkoetter P.T., Bork T., Salou S., Liang W., Mizi A., Özel C., Koehler S., Hagmann H.H., Ising C., Kuczkowski A. (2019). Anaerobic Glycolysis Maintains the Glomerular Filtration Barrier Independent of Mitochondrial Metabolism and Dynamics. Cell Rep..

[bib5] Bruggeman L.A., Wu Z., Luo L., Madhavan S.M., Konieczkowski M., Drawz P.E., Thomas D.B., Barisoni L., Sedor J.R., O'Toole J.F. (2016). APOL1-G0 or APOL1-G2 Transgenic Models Develop Preeclampsia but Not Kidney Disease. J. Am. Soc. Nephrol..

[bib6] Chan D.C. (2020). Mitochondrial Dynamics and Its Involvement in Disease. Annu. Rev. Pathol..

[bib7] Chaudhry A., Shi R., Luciani D.S. (2020). A pipeline for multidimensional confocal analysis of mitochondrial morphology, function, and dynamics in pancreatic β-cells. Am. J. Physiol. Endocrinol. Metab..

[bib8] Chen T.K., Tin A., Peralta C.A., Appel L.J., Choi M.J., Lipkowitz M.S., Winkler C.A., Estrella M.M. (2017). APOL1 Risk Variants, Incident Proteinuria, and Subsequent eGFR Decline in Blacks with Hypertension-Attributed CKD. Clin. J. Am. Soc. Nephrol..

[bib9] Chun J., Riella C.V., Chung H., Shah S.S., Wang M., Magraner J.M., Ribas G.T., Ribas H.T., Zhang J.Y., Alper S.L. (2022). DGAT2 Inhibition Potentiates Lipid Droplet Formation To Reduce Cytotoxicity in APOL1 Kidney Risk Variants. J. Am. Soc. Nephrol..

[bib10] Cuypers B., Lecordier L., Meehan C.J., Van den Broeck F., Imamura H., Büscher P., Dujardin J.C., Laukens K., Schnaufer A., Dewar C. (2016). Apolipoprotein L1 Variant Associated with Increased Susceptibility to Trypanosome Infection. mBio.

[bib11] Daneshpajouhnejad P., Kopp J.B., Winkler C.A., Rosenberg A.Z. (2022). The evolving story of apolipoprotein L1 nephropathy: the end of the beginning. Nat. Rev. Nephrol..

[bib12] Freedman B.I., Julian B.A., Pastan S.O., Israni A.K., Schladt D., Gautreaux M.D., Hauptfeld V., Bray R.A., Gebel H.M., Kirk A.D. (2015). Apolipoprotein L1 gene variants in deceased organ donors are associated with renal allograft failure. Am. J. Transplant..

[bib13] Freedman B.I., Kopp J.B., Sampson M.G., Susztak K. (2021). APOL1 at 10 years: progress and next steps. Kidney Int..

[bib14] Friedman D.J., Pollak M.R. (2011). Genetics of kidney failure and the evolving story of APOL1. J. Clin. Investig..

[bib15] Genovese G., Friedman D.J., Ross M.D., Lecordier L., Uzureau P., Freedman B.I., Bowden D.W., Langefeld C.D., Oleksyk T.K., Uscinski Knob A.L. (2010). Association of trypanolytic ApoL1 variants with kidney disease in African Americans. Science.

[bib16] Granado D., Müller D., Krausel V., Kruzel-Davila E., Schuberth C., Eschborn M., Wedlich-Söldner R., Skorecki K., Pavenstädt H., Michgehl U., Weide T. (2017). Intracellular APOL1 Risk Variants Cause Cytotoxicity Accompanied by Energy Depletion. J. Am. Soc. Nephrol..

[bib17] Hale L.J., Howden S.E., Phipson B., Lonsdale A., Er P.X., Ghobrial I., Hosawi S., Wilson S., Lawlor K.T., Khan S. (2018). 3D organoid-derived human glomeruli for personalised podocyte disease modelling and drug screening. Nat. Commun..

[bib18] Itoku A., Isaac J., Wilson S., Reidy K., Kaskel F. (2024). APOL1 Nephropathy Risk Variants Through the Life Course: A Review. Am. J. Kidney Dis..

[bib19] Juliar B.A., Stanaway I.B., Sano F., Fu H., Smith K.D., Akilesh S., Scales S.J., El Saghir J., Bhatraju P.K., Liu E. (2024). Interferon-γ induces combined pyroptotic angiopathy and APOL1 expression in human kidney disease. Cell Rep..

[bib20] Koning M., Dumas S.J., Avramut M.C., Koning R.I., Meta E., Lievers E., Wiersma L.E., Borri M., Liang X., Xie L. (2022). Vasculogenesis in kidney organoids upon transplantation. NPJ Regen. Med..

[bib21] Koning M., van den Berg C.W., Rabelink T.J. (2020). Stem cell-derived kidney organoids: engineering the vasculature. Cell. Mol. Life Sci..

[bib22] Kopp J.B., Nelson G.W., Sampath K., Johnson R.C., Genovese G., An P., Friedman D., Briggs W., Dart R., Korbet S. (2011). APOL1 genetic variants in focal segmental glomerulosclerosis and HIV-associated nephropathy. J. Am. Soc. Nephrol..

[bib23] Langefeld C.D., Comeau M.E., Ng M.C.Y., Guan M., Dimitrov L., Mudgal P., Spainhour M.H., Julian B.A., Edberg J.C., Croker J.A. (2018). Genome-wide association studies suggest that APOL1-environment interactions more likely trigger kidney disease in African Americans with nondiabetic nephropathy than strong APOL1-second gene interactions. Kidney Int..

[bib24] Larsen C.P., Beggs M.L., Saeed M., Ambruzs J.M., Cossey L.N., Messias N.C., Walker P.D., Freedman B.I. (2015). Histopathologic findings associated with APOL1 risk variants in chronic kidney disease. Mod. Pathol..

[bib25] Larsen C.P., Wickman T.J., Braga J.R., Matute-Trochez L.A., Hasty A.E., Buckner L.R., Arthur J.M., Haun R.S., Velez J.C.Q. (2021). APOL1 Risk Variants and Acute Kidney Injury in Black Americans with COVID-19. Clin. J. Am. Soc. Nephrol..

[bib26] Liu E., Radmanesh B., Chung B.H., Donnan M.D., Yi D., Dadi A., Smith K.D., Himmelfarb J., Li M., Freedman B.S., Lin J. (2020). Profiling APOL1 Nephropathy Risk Variants in Genome-Edited Kidney Organoids with Single-Cell Transcriptomics. Kidney360.

[bib27] Luong M.X., Auerbach J., Crook J.M., Daheron L., Hei D., Lomax G., Loring J.F., Ludwig T., Schlaeger T.M., Smith K.P. (2011). A call for standardized naming and reporting of human ESC and iPSC lines. Cell Stem Cell.

[bib28] Ma L., Ainsworth H.C., Snipes J.A., Murea M., Choi Y.A., Langefeld C.D., Parks J.S., Bharadwaj M.S., Chou J.W., Hemal A.K. (2020). APOL1 Kidney-Risk Variants Induce Mitochondrial Fission. Kidney Int. Rep..

[bib29] Ma L., Chou J.W., Snipes J.A., Bharadwaj M.S., Craddock A.L., Cheng D., Weckerle A., Petrovic S., Hicks P.J., Hemal A.K. (2017). APOL1 Renal-Risk Variants Induce Mitochondrial Dysfunction. J. Am. Soc. Nephrol..

[bib30] McCann K.J., Christensen S.M., Colby D.H., McGuire P.J., Myles I.A., Zerbe C.S., Dalgard C.L., Sukumar G., Leonard W.J., McCormick B.A., Holland S.M. (2022). IFNgamma regulates NAD+ metabolism to promote the respiratory burst in human monocytes. Blood Adv..

[bib31] McNulty M.T., Fermin D., Eichinger F., Jang D., Kretzler M., Burtt N.P., Pollak M.R., Flannick J., Weins A., Friedman D.J. (2022). A glomerular transcriptomic landscape of apolipoprotein L1 in Black patients with focal segmental glomerulosclerosis. Kidney Int..

[bib32] Murphy C., Feifel E., Jennings P., Gstraunthaler G., Wilmes A. (2019). A Protocol for One-Step Differentiation of Human Induced Pluripotent Stem Cells into Mature Podocytes. Methods Mol. Biol..

[bib33] Murphy C., Jennings P., Wilmes A. (2024). Transcriptomic profile of human iPSC-derived podocyte-like cells exposed to a panel of xenobiotics. Toxicol. Vitro.

[bib34] Murphy C., Naderlinger E., Mater A., Kluin R.J.C., Wilmes A. (2023). Comparison of human recombinant protein coatings and fibroblast-ECM to Matrigel for induced pluripotent stem cell culture and renal podocyte differentiation. ALTEX.

[bib35] Nishimura K., Sano M., Ohtaka M., Furuta B., Umemura Y., Nakajima Y., Ikehara Y., Kobayashi T., Segawa H., Takayasu S. (2011). Development of defective and persistent Sendai virus vector: a unique gene delivery/expression system ideal for cell reprogramming. J. Biol. Chem..

[bib36] Nystrom S.E., Li G., Datta S., Soldano K.L., Silas D., Weins A., Hall G., Thomas D.B., Olabisi O.A. (2022). JAK inhibitor blocks COVID-19 cytokine–induced JAK/STAT/APOL1 signaling in glomerular cells and podocytopathy in human kidney organoids. JCI Insight.

[bib37] O'Toole J.F., Schilling W., Kunze D., Madhavan S.M., Konieczkowski M., Gu Y., Luo L., Wu Z., Bruggeman L.A., Sedor J.R. (2018). ApoL1 Overexpression Drives Variant-Independent Cytotoxicity. J. Am. Soc. Nephrol..

[bib38] Parsa A., Kao W.H.L., Xie D., Astor B.C., Li M., Hsu C.Y., Feldman H.I., Parekh R.S., Kusek J.W., Greene T.H. (2013). APOL1 risk variants, race, and progression of chronic kidney disease. N. Engl. J. Med..

[bib39] Rauch C., Feifel E., Kern G., Murphy C., Meier F., Parson W., Beilmann M., Jennings P., Gstraunthaler G., Wilmes A. (2018). Differentiation of human iPSCs into functional podocytes. PLoS One.

[bib40] Reeves-Daniel A.M., DePalma J.A., Bleyer A.J., Rocco M.V., Murea M., Adams P.L., Langefeld C.D., Bowden D.W., Hicks P.J., Stratta R.J. (2011). The APOL1 gene and allograft survival after kidney transplantation. Am. J. Transplant..

[bib41] Ryu J.H., Ge M., Merscher S., Rosenberg A.Z., Desante M., Roshanravan H., Okamoto K., Shin M.K., Hoek M., Fornoni A., Kopp J.B. (2019). APOL1 renal risk variants promote cholesterol accumulation in tissues and cultured macrophages from APOL1 transgenic mice. PLoS One.

[bib42] Scales S.J., Gupta N., De Mazière A.M., Posthuma G., Chiu C.P., Pierce A.A., Hötzel K., Tao J., Foreman O., Koukos G. (2020). Apolipoprotein L1-Specific Antibodies Detect Endogenous APOL1 inside the Endoplasmic Reticulum and on the Plasma Membrane of Podocytes. J. Am. Soc. Nephrol..

[bib43] Shah S.S., Lannon H., Dias L., Zhang J.Y., Alper S.L., Pollak M.R., Friedman D.J. (2019). APOL1 Kidney Risk Variants Induce Cell Death via Mitochondrial Translocation and Opening of the Mitochondrial Permeability Transition Pore. J. Am. Soc. Nephrol..

[bib44] Thomson R., Genovese G., Canon C., Kovacsics D., Higgins M.K., Carrington M., Winkler C.A., Kopp J., Rotimi C., Adeyemo A. (2014). Evolution of the primate trypanolytic factor APOL1. Proc. Natl. Acad. Sci. USA.

[bib45] van den Berg C.W., Ritsma L., Avramut M.C., Wiersma L.E., van den Berg B.M., Leuning D.G., Lievers E., Koning M., Vanslambrouck J.M., Koster A.J. (2018). Renal Subcapsular Transplantation of PSC-Derived Kidney Organoids Induces Neo-vasculogenesis and Significant Glomerular and Tubular Maturation In Vivo. Stem Cell Rep..

[bib46] Vanhamme L., Paturiaux-Hanocq F., Poelvoorde P., Nolan D.P., Lins L., Van Den Abbeele J., Pays A., Tebabi P., Van Xong H., Jacquet A. (2003). Apolipoprotein L-I is the trypanosome lytic factor of human serum. Nature.

[bib47] Wang G., Heijs B., Kostidis S., Mahfouz A., Rietjens R.G.J., Bijkerk R., Koudijs A., van der Pluijm L.A.K., van den Berg C.W., Dumas S.J. (2022). Analyzing cell-type-specific dynamics of metabolism in kidney repair. Nat. Metab..

[bib48] Wang G., Heijs B., Kostidis S., Rietjens R.G.J., Koning M., Yuan L., Tiemeier G.L., Mahfouz A., Dumas S.J., Giera M. (2022). Spatial dynamic metabolomics identifies metabolic cell fate trajectories in human kidney differentiation. Cell Stem Cell.

[bib49] Weckerle A., Snipes J.A., Cheng D., Gebre A.K., Reisz J.A., Murea M., Shelness G.S., Hawkins G.A., Furdui C.M., Freedman B.I. (2016). Characterization of circulating APOL1 protein complexes in African Americans. J. Lipid Res..

[bib50] Wu J., Ma Z., Raman A., Beckerman P., Dhillon P., Mukhi D., Palmer M., Chen H.C., Cohen C.R., Dunn T. (2021). APOL1 risk variants in individuals of African genetic ancestry drive endothelial cell defects that exacerbate sepsis. Immunity.

[bib51] Youle R.J., van der Bliek A.M. (2012). Mitochondrial fission, fusion, and stress. Science.

[bib52] Zhang Z., Sun Z., Fu J., Lin Q., Banu K., Chauhan K., Planoutene M., Wei C., Salem F., Yi Z. (2021). Recipient APOL1 risk alleles associate with death-censored renal allograft survival and rejection episodes. J. Clin. Investig..

