# Supplementary material for: APOL1 risk variants induce metabolic reprogramming of podocytes in patient-derived kidney organoids
Source: Stem Cell Reports. 2025 Oct 2;20(10):102650. doi: 10.1016/j.stemcr.2025.102650 (PMC12790724; doi:10.1016/j.stemcr.2025.102650)
Supplement: Document S1. Figures S1–S5, Tables S2–S5, and supplemental methods [file mmc1.pdf]

Stem Cell Reports, Volume *20*

Supplemental Information

APOL1 risk variants induce metabolic reprogramming of podocytes in patient-derived kidney organoids

Heein Song, Sébastien J. Dumas, Gangqi Wang, Lijun Ma, Franca Witjas, M. Cristina Avramut, Cathelijne W. van den Berg, Michael V. Rocco, Barry I. Freedman, Ton J. Rabelink, and H. Siebe Spijker

Supplemental information

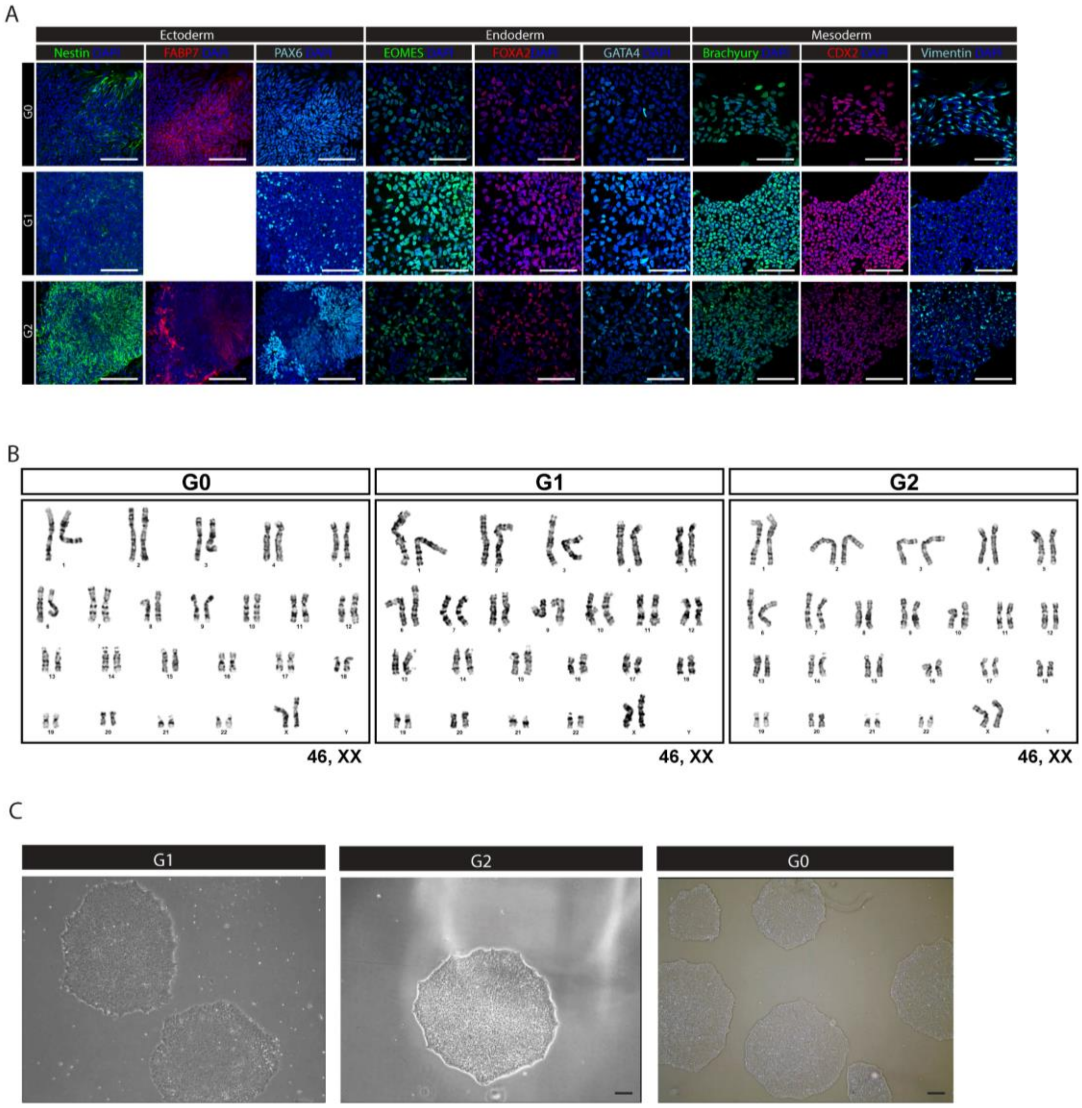

Figure S1: iPSC line characterization.

A Immunostaining images of ectoderm markers (Nestin, FABP7, PAX6), endoderm markers (EOMES, FOXA2, GATA4) and mesoderm markers (Brachyury, CDX2, Vimentin) in G0, G1, G2 iPSC lines. Scale bar, 100µm **B** Isogenic control iPSCs, G1 patient iPSCs and G2 patient iPSCs show normal karyotype of 46, XX. **C** Brightfield images showing normal iPSC morphology of G0, G1, G2 iPSCs. Scale bar, 200µm.

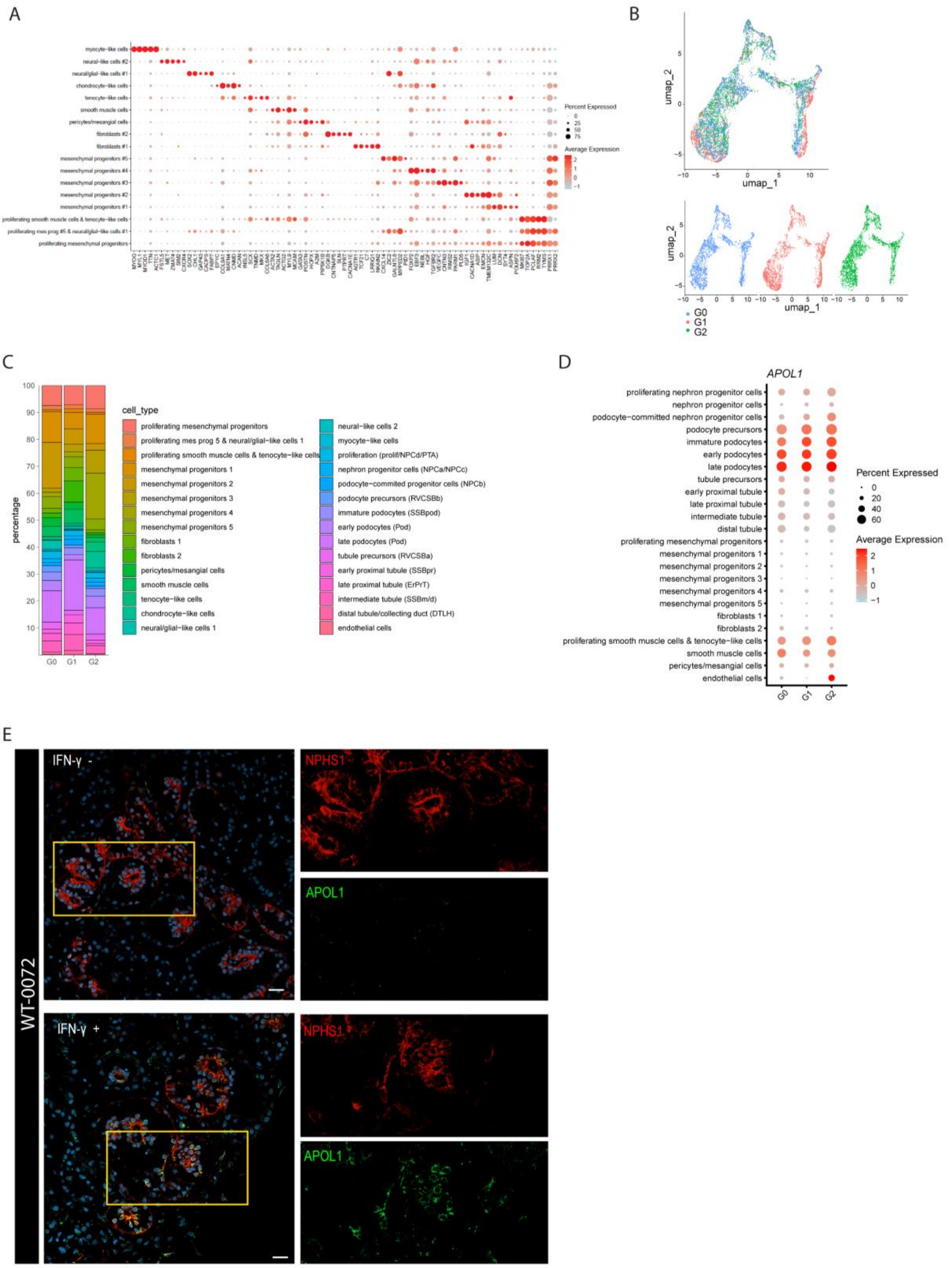

Figure S2: Non-nephron cell clustering and APOL1 expression in podocytes

A Dot plot showing marker gene expression in mesenchymal cell clusters. **B** UMAP visualization of nephron cells obtained from G0, G1, and G2 iPSC-derived kidney organoids at d7+20. Color-coded by iPSC line. **C** Relative cluster quantification for all cell types of kidney organoid from each iPSC

line, showing similar proportions of cell populations between RV and G0 organoids. **D** Dot plot presenting *APOL1* expression in all cell clusters. **E** Immunostaining shows *APOL1* expression in non-isogenic control iPSC derived kidney organoids is also mainly co-localized with NPHS1$^+$ (podocyte) cells. Scale bar, 20 µm.

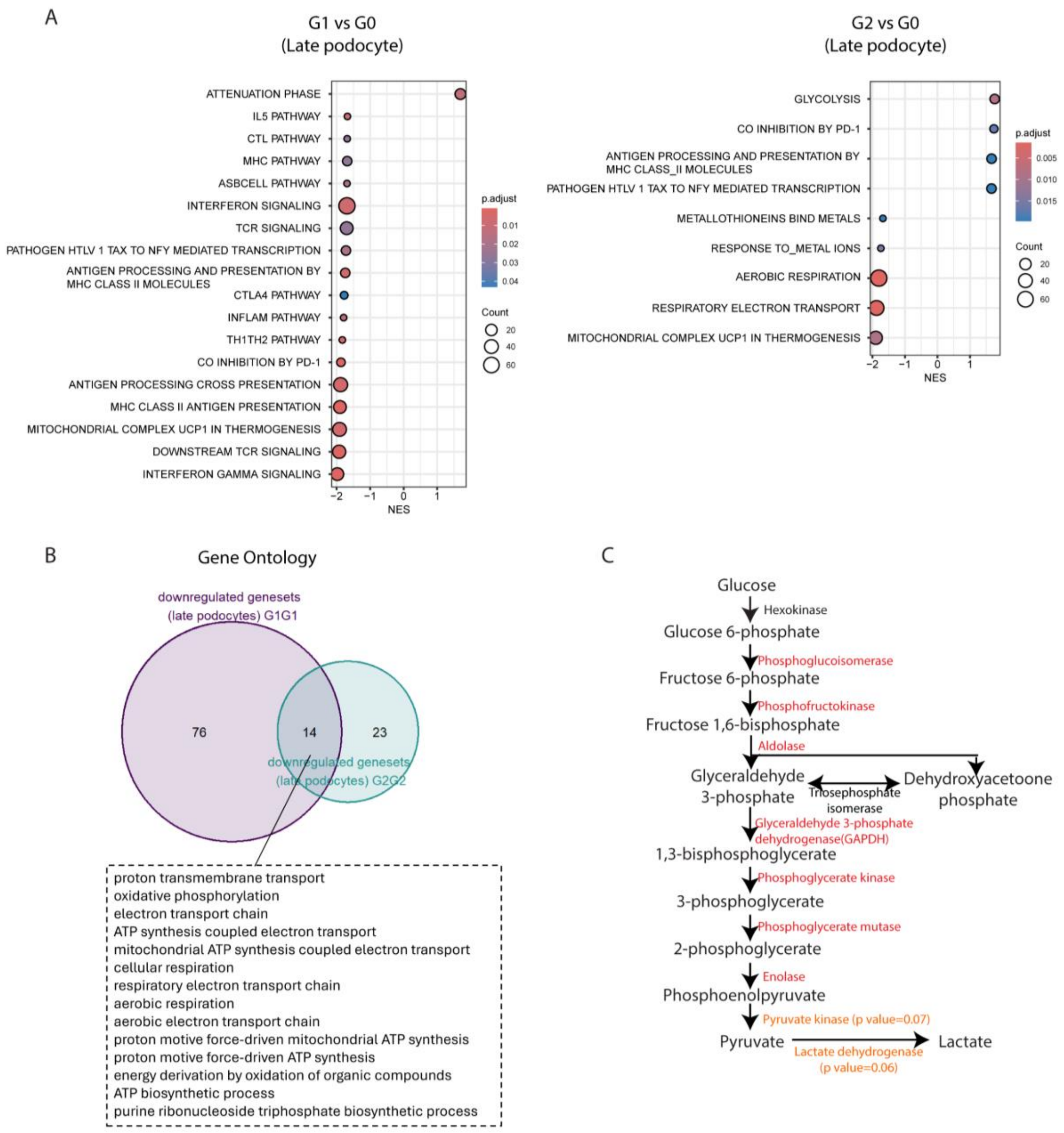

Figure S3: Separate gene set enrichment analysis (GSEA) for G1, G2, and G0 late podocytes, and positive correlation of glycolytic enzyme genes with high risk *APOL1* variants from the NEPTUNE cohort study.

A Gene set enrichment analysis (GSEA) using canonical pathway for G1 vs G0 and G2 vs G0 late podocytes. NES: normalized enrichment score. **B** Overlapping downregulated genesets between G1 and G2 compared to G0, using gene ontology (GO) database. **C** Glycolytic enzyme genes that show significant positive correlation with high risk APOL1 variants are marked as red.

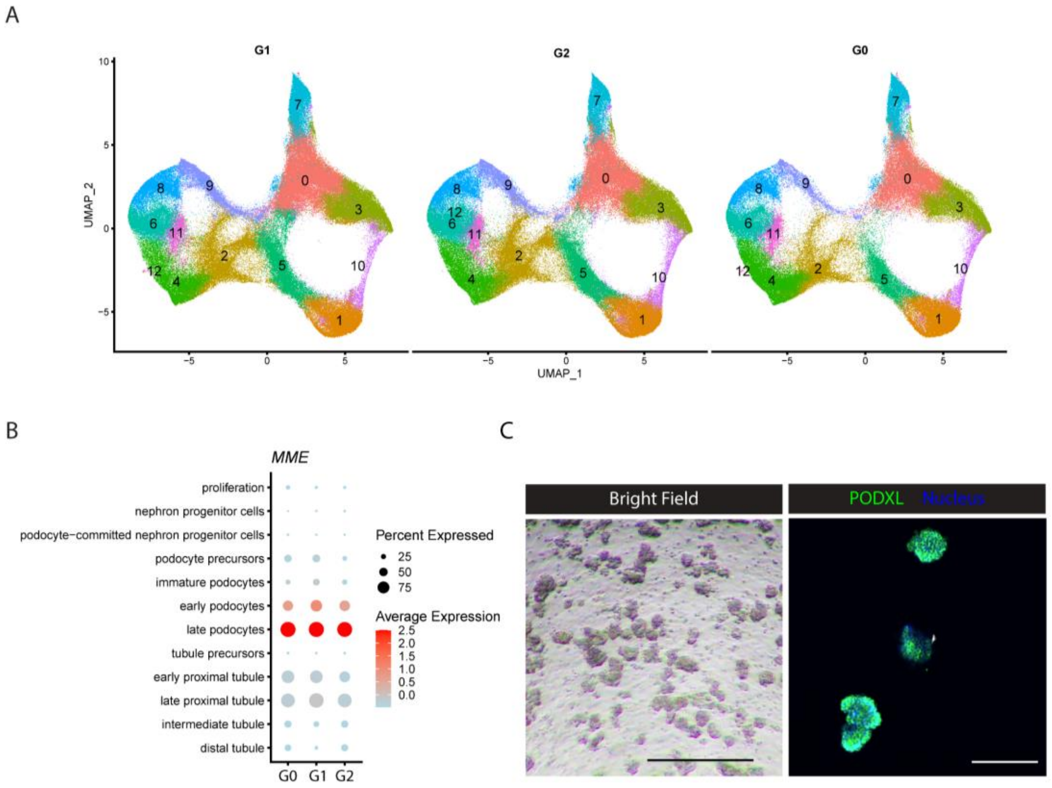

Figure S4: Organoid cell clustering based on lipid profile and the isolation of late podocytes.

A Separate UMAP visualization of integrated lipidomics data of d7+20 kidney organoids from G0, G1, and G2 iPSCs, showing similar clustering between 3 genotypes. **B** Dot plot from single cell RNA transcriptomics shows MME is highly expressed in late podocyte cluster. **C** Bright field image shows glomeruli sieved from dissociated kidney organoids. and immunostaining image showing that Isolated gloms are PODXL^{+} . Scale bar, 100 µm.

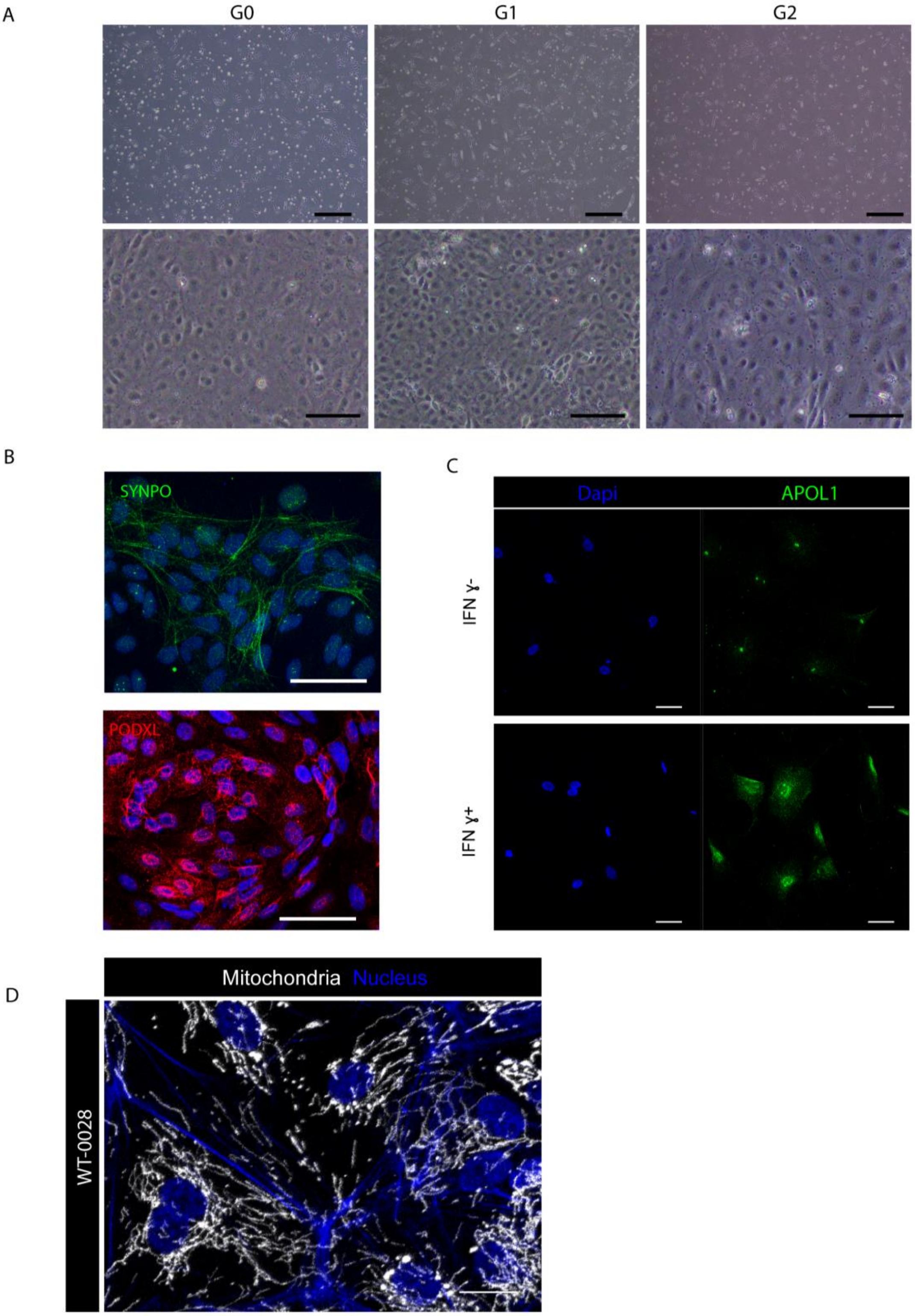

Figure S5: iPSC derived podocyte differentiation.

A Bright field image showing day 1 (upper panel, scale bar, 500 μm) and day 10 (lower panel, scale bar, 100 μm) of podocyte differentiation. **B** Differentiated podocytes were positive with Synaptopodin and PODXL. Scale bar, 50 μm. **C** Representative confocal image showing APOL1 expression in

iPSC-derived podocytes upon IFN-γ treatment. Scale bar, 50 μm. **D** Representative image of normal mitochondrial morphology from non-isogenic control G0 podocyte after IFN-γ treatment. Scale bar, 20 μm.

Table S1 Significantly upregulated or downregulated genes in G1+G2 compared to G0, G1 vs G0, and G2 vs G0. Log2 Fold change(Log2FC) threshold set at 0.2 and -0.2. Adjusted p-value<0.05.

Table S2 Genome-wide APOL1 correlation plots of glycolysis genes, based on glomerular transcriptome data from the NEPTUNE cohort study(PMID 34929253).

**Gene Symbol**	**Low-risk Correlation**	**Low-risk P-value**	**High-risk Correlation**	**High-risk P-value**
HK1	0.39	0.17	0.29	0.28
HK2	0.33	0.25	0.16	0.56
GPI	-0.05	0.87	0.54	0.03
PFKM	0.01	0.98	0.61	0.0142
PFKP	0.49	0.08	0.57	0.02
PFKL	0.38	0.18	0.38	0.14
PFKFB2	-0.17	0.56	0.31	0.23
PFKFB3	0.05	0.86	0.11	0.69
PFKFB4	0.39	0.17	0.21	0.42
ALDOA	-0.58	0.03	0.51	0.05
ALDOB	-0.51	0.06	-0.12	0.66
ALDOC	-0.14	0.63	-0.1	0.71
TPI1	-0.68	7.20e-3	0.18	0.51
GAPDH	-0.59	0.03	0.77	4.48e-4
PGK1	-0.1	0.73	0.73	1.93e-3
PGAM1	-0.44	0.12	0.74	1.50e-3
ENO1	-0.31	0.27	0.61	0.0131
PKM	0.17	0.57	0.47	0.07
LDHA	0.01	0.99	0.49	0.06

Table S3 Oligonucleotides used for qRT-PCR, DNA sequencing and gene editing

		Forward primer (5'-3')	Reverse primer (5'-3')
qPCR	APOL1	TGATAATGAGGCCTGGAACG	TACTGCTGGCCTTTATCGTG
	S18	GCAGAATCCACGCCAGTACAAG	GCTTGTTGTCCAGACCATTGGC
DNA sequencing	APOL1	ACCAACTCACACGAGGCATT	CACCATTGCACTCCAACTTGGC
		Sequence	
APOL1 risk variant isogenic control generation	gRNA	5’- TCTCAACAATAAGATTCTGC-3’	
	ssODN	5’-GGAGCTGAAGAAGGTGGCTCAGGAGCTGGAGGAGAAGCTAAAC ATTCTCAACAATAATTATAAGATTCTGCAGGCGGACCAAGAACTGTGACC ACAGGGCAGGGCAGCCACCAGGAGAGA-3’	

Table S4 iPSC lines used

**iPSC line**	**Nomenclature in the manuscript**	***APOL1* genotype**
LUMC0214iAPOL	G1	G1G1
LUMC0216iAPOL	G2	G2G2
ISO01LUMC0216iAPOL05	G0	G0G0
ISO03LUMC0216iAPOL05	G0	G0G0
LUMC0072iCTRL01	WT-0072	G0G0
iPSC0028	WT-0028	G0G0

Table S5 Immunostaining antibody list

**Primary antibody**	**Source**	**Identifier**
Sheep anti-NEPHRIN1	R&D	AF4269
LTL-biotin-conjugated	Vector Laboratories	B-1325
Mouse anti-ECAD	BD Biosciences	610181
Rabbit anti-APOL1	Abcam	ab108315
Goat anti-PODOCALYXIN	R&D	AF1658
Rabbit anti-MME	Proteintech	23782-1-AP
Mouse anti-mitochondria	Abcam	Ab92824
Oct3/4-BV421	BDbiosciences	565644
Nanog-PE	BDbiosciences	560483
SSEA4-FITC	Miltenyi	130-098-371
**Secondary antibody**		
Donkey anti-Sheep IgG-Alexa Fluor 568	Thermo Fisher Scientific	A21099
Streptavidin, Alexa Fluor 647 conjugate	Thermo Fisher Scientific	S21374

Donkey anti-Mouse IgG-Alexa Fluor 488	Thermo Fisher Scientific	A21202
Donkey anti-Mouse IgG-Alexa Fluor 647	Thermo Fisher Scientific	A31571
Donkey anti-Rabbit IgG-Alexa Fluor 488	Thermo Fisher Scientific	A21206
Donkey anti-Goat IgG-Alexa Fluor 568	Thermo Fisher Scientific	A11057
**Ectoderm, Endoderm, Mesoderm markers**		
(All antibodies below have been custom conjugated by Cell signaling technologies. The Clone and Ordering number for primary antibodies are as below)		
Nestin (10C2) mouse mAB - Alexa 488		#33475
FABP7 (D8N3N)) Rabbit mAB - Alexa 555		#13347
Pax6 (D3A9V) Rabbit mAB - Alexa 647		#60433
EOMES (D8D1R) Rabbit mAB - Alexa 488		#81493
FOXA2 D56D6) Rabbit mAB - Alexa 555		#8186
GATA4 (D3A3M) Rabbit mAB - Alexa 647		#36966
Brachyury (D2Z3J) Rabbit mAB - Alexa 647		#81694
CDX2 (D11D10) Rabbit mAB -- Alexa 555		#12306
Vimentin(D21H3) Rabbit mAB - Alexa 647		#9856

Supplemental methods

Sequencing analysis

Genomic DNA was extracted from iPSCs using NucleoSpin Tissue kit (Bioké). The primer sequences used for standard polymerase chain reaction (PCR) are listed in Table S3. After amplification, PCR products were purified using Wizard SV Gel and PCR Clean-Up System (Promega). Sequences were processed on Applied Biosystems 96 capillaries (ABI3730xl).

iPSC culture and organoid differentiation

hiPSCs were maintained with Essential 8 medium (E8, Thermo Fisher Scientific) on vitronectin and differentiated using previously reported protocols(Takasato et al., 2016; van den Berg et al., 2018). hiPSCs were passaged using 0.5mM EDTA solution, pH 8.0 (Invitrogen, 15575-020, diluted in PBS) and stored in liquid N_2 below passage 20. hiPSCs were confirmed to be mycoplasma free and maintained below passage 50 during the experiments. hiPSCs were plated for differentiation in E8 medium supplemented with RevitaCell for 24hr (day -1). Cells were cultured for 4 days in 8 μM CHIR99021 (R&D Systems) in STEMdiff APEL2 medium (Stem Cell Technologies) supplemented with 1% Protein Free Hybridoma Medium II (PFHMII, Thermo Fisher Scientific) and Antibiotic-Antimycotic (Thermo Fisher Scientific). From day 4, cells were incubated with 200ng/mL rhFGF9 (R&D Systems) and 1ug/mL heparin (Sigma Aldrich) in STEMdiff APEL2 medium. At day 7, cells were transferred from monolayer culture to 3D culture on Transwell 0.4-μm pore polyester membranes and maintained for 5 days in STEMdiff APEL2 medium supplemented with 200 ng mL-1 rhFGF9 and 1 μg mL-1 heparin. For the remaining days organoids were refreshed every other day with STEMdiff APEL2 without FGF9 and heparin.

iPSC-derived podocyte differentiation

iPSCs were passaged into a single cell suspension using TryPLE (Thermo Fisher Scientific) and centrifuged at 300g for 5min. The cells were resuspended in Podocyte differentiation medium consisting of DMEM/HAM-F-12 (Gibco), non-essential amino acids (Gibco), Glutamax (Gibco), Insulin-Transferrin-Sodium selenite media supplement (Sigma Aldrich), supplemented with 1.25% FBS, 100 U/mL Penicillin-Streptomycin and growth factors: 15 ng/mL BMP7 (Gibco, PHC9544), 10 ng/ml Activin A (Stemcell Technology, 78001) and 100nM retinoic acid (Stemcell Technology, 72262). The cells were seeded at a density of 20,000 cells per cm^2 along with 10 μM Pho-associated kinase (ROCK) inhibitor Y-27632. The podocyte differentiation medium was refreshed on day 1, 3, 6,

and 9. From day 10 onwards, the growth factors were removed from the podocyte differentiation medium and refreshed every other day.

Immunofluorescence analysis

Kidney organoids were fixed with 2% PFA for 20min or quickly frozen in liquid nitrogen. Paraffin embedded kidney organoids were sectioned with microtome at 4μm. Following rehydration of the tissue sections, antigen retrieval was done in 10mM sodium citrate buffer (pH6.0). Frozen kidney organoids were embedded in OCT and sectioned at the cryotome at 4μm. Frozen sections were fixed with 2% PFA for 10min at room temperature and permeabilized with 0.3% Triton X in PBS for 15min at room temperature. Antigen retrieval with 10mM sodium citrate buffer (pH6.0) was done when necessary. Sections were incubated overnight at 4 °C with primary antibodies, followed by incubation with secondary antibody.

For podocyte cell immunofluorescence staining, cells were fixed with 4% PFA for 15min and blocked with 5% normal donkey serum in 0.3% Triton X in PBS for 50 min at room temperature. Podocytes were incubated overnight at 4 °C with primary antibodies, followed by incubation with secondary antibody. Samples were counterstained with Hoechst33258 (Thermo Fisher Scientific) and embedded in ProLong Gold Antifade Mountant (Thermo Fisher Scientific). Organoid sections and podocyte cells were examined using the Zeiss LSM 900 Airyscan confocal microscope and Leica White Light Laser Confocal Microscope TCS SP8. The antibodies used in this study are listed in Table S5.

mRNA isolation and qPCR

Total RNA was extracted using nucleospin RNA/protein kit (Bioké) according to the manufacturer's protocol. SuperScript III Reverse Transcriptase (Invitrogen) was used for cDNA synthesis. qPCR was performed with SYBR Green Supermix (Bio-Rad) and the CFX Connect Real-Time System (Bio-Rad) was used. The expression of genes was determined by normalization to 18S levels. Primers used are listed in Supplementary Table S3.

Organoid dissociation to single cells

5 organoids from each cell line were placed in Collagenase I buffer consisting of 600U/mL collagenase Type 1 (Worthington) and 0.75 U/mL DNAse (Sigma Aldrich) in HBSS with calcium and magnesium (Thermo Fisher Scientific) and incubated in a water bath at 37 °C for 40min with pipetting up and down 2-3 times with 1mL pipet. The cell suspensions were centrifuged at 300g for 7min and the supernatant was removed. Cells were resuspended in TryPLE buffer consisting of

5U/mL DNAse I (Sigma Aldrich) and 4 ug/mL heparin (Sigma Aldrich) in 80% TrypLE select 10x (Thermo Fisher Scientific) in DPBS (Thermo Fisher Scientific) and incubated at 37 °C for 5 min with repeat pipetting. The dissociation was stopped by adding cold HBSS with 10% FCS. The single cell suspension was centrifuged at 400g and the cell pellet resuspended in PBS with 0.1% BSA.

Single cell RNA sequencing data analysis

Single cell data preprocessing: Seurat objects were created for each sample using *CreateSeuratObject()* function retaining genes expressed in at least 3 cells and cells expressing at least 200 genes, and merged using the *merge()* function (total cell number: 27,116 cells). Doublets were identified using the scDblFinder package (v1.16.0). To do so, data were log-normalized using *NormalizeData()* function, and the 2,000 most variable genes were identified and scaled using *FindVariableFeatures()* and *ScaleData()* functions, respectively. Dimensionality reduction was performed using *RunPCA()* and *RunUMAP()* functions using the 20 first PCs, a number determined from the Elbow plot of standard deviations of each PC. Data were clustered using *FindNeighbors()* function on the 20 first PCs with "k.param" argument set at 30, and *FindClusters()* with resolution set at 0.5 and all other options as default. Doublets were identified specifying sample identities and obtained clusters, with the *scDblFinder()* function, and then removed (2,606 doublets identified). Low quality cells expressing less than 1,500 genes/cell were further removed, as well as cells with high mitochondrial gene fraction (>7%) and low ribosomal gene fraction (<4%), leading to a final total number of 20,174 high-quality cells retained for downstream analyses (5,518 detected genes per cell in average).

Single cell data analysis: The 2,000 most highly variable features were identified and scaled as described above. Dimensionality reduction was performed with the *RunPCA()* and *RunUMAP()* functions including the first 16 PCs and "min.dist" argument set at 0.2, and clustering using the same number of PCs for the *FindNeighbors()* function and a resolution set at 0.2 for the *FindClusters()* function. Markers genes for each cluster were identified using *FindAllMarkers()* functions with "only.pos" argument set TRUE. The main populations, mesenchymal cells, podocytes, tubular epithelial cells, nephron progenitor/precursors, and endothelial cells were identified according to previously described canonical marker genes (*PECAM1, CDH5 , KDR , ICAM2 , ESM1* for endothelial cells, *LYPD1, DAPL1, CDH6, LAMP5, PAX2* for nephron progenitors/precursors, *EPCAM, FXYD2, APOE, SPP1, CUBN* for tubular epithelial cells, *NPHS1, NPHS2, PODXL, WT1, MAFB* for podocytes, and *COL1A1, COL2A1, COL3A1, CRABP1, PRRX1* for mesenchymal cells). Nephron cells (including podocytes, tubular epithelial cells, and nephron progenitor/precursors) and mesenchymal cells were subset separately for in-depth clustering. Identification of highly variable features, scaling, dimensionality reduction, and clustering was performed as described above with minor modifications (resolution set at 1 for *FindClusters()* function, and the first 17 PCs used for the *RunUMAP()* and

FindNeighbors() functions for mesenchymal cells). Clusters representing biologically similar cell phenotypes were merged and annotated according to the top marker genes and previous knowledge on kidney organoid cell heterogeneity(Koning et al., 2022). Heterogeneity of the "late podocyte" cluster was investigated subsetting this cell population. Batch-correction for differences in cell lines using CCA method as implemented in Seurat package was performed, since this effect was otherwise captured (late podocytes from G1 organoids clustering separately from their G0 and G2 counterparts). Specifically, the *FindIntegrationAnchors()* function was used with the first 11 PCs and "anchor.features" argument set at 2,000, and the resulting identified anchors were provided for the integration using *IntegrateData()* function on the same number of PCs. Integrated data were scaled, and dimensionality reduction performed as described above using the first 11 PCs for calculating UMAP with "min.dist" argument set at 0.1. Clustering was performed as described above with resolution set at 0.2 resulting in 4 clusters. Cluster annotation was performed according to the marker gene signature of each cluster, identified with *FindAllMarkers()* function.

Umap and dot plot visualization: Umap plots were generated using *DimPlot()* function from the Seurat package. When needed, the argument "split.by" set to metadata value "genotype" was used to plot cells from G0, G1 and G2 organoids separately. Dot plots were prepared using *DotPlot()* function as implemented in Seurat. Normalized and scaled gene expression values were displayed.

Metadata quantification, analysis and visualization: metadata were extracted from Seurat objects using *table()* function selecting the metadata of interest. Percentage was calculated for each cluster per organoid sample and displayed as bar plot generated with the *geom_bar()* function from the ggplot2 package (v3.4.4). Statistical analysis to assess differences in cluster proportions between *APOL1* RV and wild type *APOL1* samples was performed using the *propeller()* function from the speckle package (v1.2.0)(Phipson et al., 2022), specifying clusters, samples and groups (RV or wild type *APOL1*) as arguments and other settings as default.

Differential gene expression analysis: To determine the number of dysregulated genes between *APOL1* RV and wildtype *APOL1* in each nephron cluster, *FindMarkers()* function from Seurat package was used with MAST statistical test, "logfc.threshold" and "min.pct" arguments set at 0, and "nCount_RNA" as well as "cell_line" as latent covariates for correcting for unequal sequencing depth and cell line difference effects. For separate *APOL1* G1 and G2 versus G0 analyses, "cell-line" metadata was not included as latent covariate. Genes were considered dysregulated when log2FC was superior at 0.2 for upregulated genes or inferior at -0.2 for downregulated genes, and when adjusted *p*-value was inferior at 0.05. Numbers of dysregulated genes for each nephron cluster were calculated and visualized as barplot generated using the *geom_bar()* function the ggplot2 package. Output of the differential gene expression analysis was also visualized for the late podocyte cluster as volcano plot using the function

EnhancedVolcano() function with "max.overlap" argument set at 60, from the EnhancedVolcano package (v1.20.0).

Gene set enrichment analysis: Results from the differential gene expression analysis between late podocytes from *APOL1* RV vs wild type *APOL1* was used as input for gene set enrichment analysis using the clusterProfiler package (v4.10.0)(Yu et al., 2012). Briefly, gene set enrichment analysis was performed with the *GSEA()* function using 2,799 curated genesets for canonical pathways (REACTOME, BIOCARTA, PID and KEGG_MEDICUS) downloaded from the molecular signature database (MSigDB)(Subramanian et al., 2005). Results were visualized using the *dotplot()* function with normalized enrichment score used as x axis.

Correlation plots for human biopsy samples from the NEPTUNE cohort: Correlation plots displaying high RV and low RV *APOL1* expression and glycolytic gene expression were generated and downloaded from the online APOL1 portal(McNulty et al., 2022).

Isolation of glomeruli from kidney organoids

A minimum of 9 kidney organoids were dissociated and glomeruli isolated according to a published protocol(Hale et al., 2018). Briefly, organoids were incubated with TrypLE select enzyme for 12min at 37'C with gentle mixing using 1ml pipette applied every 3 min. After dissociation, the cell solution was added to a 70μm cell strainer and flow through was collected. Subsequently, the suspension was applied to a 40μm cell strainer and glomeruli were collected by retrieving from the sieve. The flow through was applied to a 30μm cell strainer to collect smaller glomeruli.

Oxygen consumption rate measurement

Glomeruli isolated from kidney organoids 3 days after IFN-γ induction (day 7+20) were plated on Seahorse XF96 polystyrene tissue culture plates (Seahorse Bioscience) and incubated in freshly made warm Krebs-Ringer bicarbonate HEPES buffer with 1mM glucose, 2mM glutamine and 0.2% BSA. After 1 hour of incubation with CO2 and 1 hour incubation without CO2, plates were loaded into a XFe 96 extracellular flux analyzer (Seahorse Bioscience). Mitochondrial respiration was assayed with glucose (20 mM), oligomycin (5 μM, ATP synthase inhibitor), FCCP (4 μM, an uncoupling agent that collapses the proton gradient), and a mixture of rotenone (2 μM, Complex I inhibitor from electron transport chain) and antimycin A (2 μM, Complex III inhibitor from electron transport chain). Compounds were applied sequentially, and the oxygen consumption was measured in 3 minute periods with 3 minute mixing during each cycle.

Tissue preparation and matrix deposition

Cryopreserved tissue biopsies were embedded in 10% gelatin and cryosectioned into 10-μm-thick sections using a Cryostar NX70 cryostat (Thermo Fisher Scientific) at –20 °C. Sections were thaw-mounted onto indium-tin-oxide (ITO)-coated glass slides (VisionTek Systems) and stored at –80°C until further use. Slides were placed in a vacuum freeze-dryer for 15 minutes prior to matrix application. After drying, *N*-(1-naphthyl) ethylenediamine dihydrochloride (NEDC) (Sigma-Aldrich, UK) MALDI-matrix solution of 7 mg/mL in methanol/acetonitrile/deionized water (70/25/5% v/v/v) was applied using a HTX M3+ Sprayer™ (HTX Technologies, USA). The spray settings were: matrix concentration, 7 mg/mL NEDC; temperature, 60 °C; number of passes, 20 layers; flow rate, 80 μL/min; velocity, 1200 mm/min; track spacing, 3 mm; gas flow rate, 10 psi; and time in between passes, 30 s.

MALDI-MSI measurement

Prior to analysis, the instrument was externally calibrated using red phosphorus. Spectra were acquired with 50 laser shots per pixel at a laser repetition rate of 5 kHz. Data acquisition was performed using flexControl (Version 4.0, Bruker Daltonics) and flexImaging 5.0 (Bruker Daltonics). Sections present on the same slide were measured in a randomized order. The *m/z* features present in MALDI-TOF-MSI dataset were further used for identity assignment of metabolites and lipid species. The *m/z* values were imported into the Human Metabolome Database (https://hmdb.ca/) after re-calibration in mMass and annotated for metabolites and lipids species with an error ≤ ±20 ppm(Wishart et al., 2018). The ^{13}C-labeled peaks were selected by comparing the spectrum of control and ^{13}C-labeling experiments and annotated based on the presence of un-labeled metabolites and their theoretical *m/z* values. Peak intensities of the selected features were exported for all the measured pixels from SCiLS Lab 2016b (version 2016b, Bruker Daltonics), which were used for the following analysis. Single ion visualizations were also obtained from SCiLS Lab.

Post-MALDI-MSI staining

Following the MALDI-MSI data acquisition, excess matrix was removed by washing the slides in 100% ethanol (2×5 min), 75% ethanol (1×5 min), and 50% ethanol (1×5 min), after which tissues on the slide were fixed using 4% paraformaldehyde for 10 minutes. For immunofluorescent staining, antigen retrieval was performed with sodium citrate buffer pH6.0, and slides were blocked with 5% normal donkey serum and 0.3% Triton X-100 in PBS for 1 hour at room temperature. Primary anti-PODXL antibody (1:100, R&D Systems, AF1658), anti-MME (1:200, Proteintech, 23782-1-AP), and lotus tetragonolobus lectin (LTL, 1:300, Vector laboratories, B1325) were incubated overnight at 4 °C, followed by correspondent fluorescent-labelled secondary antibodies for 1 hour at room temperature.

Slides were embedded in Prolong gold antifade mountant with DAPI (Thermo Fisher Scientific, P36931). The stained tissues were scanned using a digital slide scanner (3D Histech Pannoramic MIDI Scanner, Sysmex). Digital scanned images were aligned with the MALDI-MSI data.

MSI data processing and analysis

For lipid analysis, features with *m/z* ≥ 400, predominately glycerophospholipids, that did not co-localize with MALDI matrix signals were selected (signal-to-noise-ratio ≥ 3). The per-pixel total ion count (TIC)-normalized intensity values for each *m/z* feature from all MSI measurements were directly exported as comma-separated values (.csv format). Upon loading in R (v. 4.0), these values were transformed into a count matrix for UMAP analysis by multiplying the intensities by 10 and taking the integer. This count data matrix was normalized and scaled using SCTransform to generate a 2-dimensional UMAP projection using Seurat(Stuart et al., 2019). To compare pixels from different samples, matrices were imported into the Seurat package and a data integration step was performed after batch correction using the method provided by Seurat. The integrated datasets were used to generate a 2-dimensional UMAP projection using Seurat and 3-dimensional UMAP projection using the Seurat and plotly packages. The spatial reconstructions of the segmentation clusters were compared to the aligned immunofluorescence staining and cell types were identified based on both immunomarker staining. Same cell types were annotated within one cluster. The differential abundance of lipids between clusters were analyzed using the FindAllMarkers function in Seurat. The embedding information of the 3-dimensional UMAP was translated to RGB color coding by varying red, green, and blue intensities on the 3 independent axes. Together with pixel coordinate information exported from SCiLS Lab, a MxNx3 matrix was generated and used to generate molecular histology images in Matlab (v. R2019a.; Mathworks).

The average peak intensity of metabolites including ^{13}C-labeling metabolites was calculated for each cluster from ^{13}C-labeling experiments. The 13C-labeled metabolite abundance was corrected to its isotope tracer purity. Natural isotope abundance correction was performed for metabolites using R package IsoCorrectoR[(Heinrich et al., 2018)]. The fraction enrichment of isotopologues was calculated based on the ratio of each 13C-labeled metabolite (isotopologue) to the sum of this metabolite abundance. The fraction enrichment of isotopologues derived from $^{13}C_5$-glutamine were further used for relative flux rate calculation according to the previous published Q-Flux equations(Hubbard et al., 2023).

Supplemental references

Hale, L. J., Howden, S. E., Phipson, B., Lonsdale, A., Er, P. X., Ghobrial, I., Hosawi, S., Wilson, S., Lawlor, K. T., Khan, S., Oshlack, A., Quinlan, C., Lennon, R., & Little, M. H. (2018). 3D organoid-derived human glomeruli for personalised podocyte disease modelling and drug screening. *Nat Commun*, *9*(1), 5167. https://doi.org/10.1038/s41467-018-07594-z

Heinrich, P., Kohler, C., Ellmann, L., Kuerner, P., Spang, R., Oefner, P. J., & Dettmer, K. (2018). Correcting for natural isotope abundance and tracer impurity in MS-, MS/MS- and high-resolution-multiple-tracer-data from stable isotope labeling experiments with IsoCorrectoR. *Sci Rep*, *8*(1), 17910. https://doi.org/10.1038/s41598-018-36293-4

Hubbard, B. T., LaMoia, T. E., Goedeke, L., Gaspar, R. C., Galsgaard, K. D., Kahn, M., Mason, G. F., & Shulman, G. I. (2023). Q-Flux: A method to assess hepatic mitochondrial succinate dehydrogenase, methylmalonyl-CoA mutase, and glutaminase fluxes in vivo. *Cell Metab*, *35*(1), 212-226.e214. https://doi.org/10.1016/j.cmet.2022.11.011

Koning, M., Dumas, S. J., Avramut, M. C., Koning, R. I., Meta, E., Lievers, E., Wiersma, L. E., Borri, M., Liang, X., Xie, L., Liu, P., Chen, F., Lin, L., Luo, Y., Mulder, J., Spijker, H. S., Jaffredo, T., van den Berg, B. M., Carmeliet, P., . . . Rabelink, T. J. (2022). Vasculogenesis in kidney organoids upon transplantation. *NPJ Regen Med*, *7*(1), 40. https://doi.org/10.1038/s41536-022-00237-4

McNulty, M. T., Fermin, D., Eichinger, F., Jang, D., Kretzler, M., Burtt, N. P., Pollak, M. R., Flannick, J., Weins, A., Friedman, D. J., & Sampson, M. G. (2022). A glomerular transcriptomic landscape of apolipoprotein L1 in Black patients with focal segmental glomerulosclerosis. *Kidney Int*, *102*(1), 136-148. https://doi.org/10.1016/j.kint.2021.10.041

Phipson, B., Sim, C. B., Porrello, E. R., Hewitt, A. W., Powell, J., & Oshlack, A. (2022). propeller: testing for differences in cell type proportions in single cell data. *Bioinformatics*, *38*(20), 4720-4726. https://doi.org/10.1093/bioinformatics/btac582

Stuart, T., Butler, A., Hoffman, P., Hafemeister, C., Papalexi, E., Mauck, W. M., 3rd, Hao, Y., Stoeckius, M., Smibert, P., & Satija, R. (2019). Comprehensive Integration of Single-Cell Data. *Cell*, *177*(7), 1888-1902.e1821. https://doi.org/10.1016/j.cell.2019.05.031

Subramanian, A., Tamayo, P., Mootha, V. K., Mukherjee, S., Ebert, B. L., Gillette, M. A., Paulovich, A., Pomeroy, S. L., Golub, T. R., Lander, E. S., & Mesirov, J. P. (2005). Gene set enrichment analysis: a knowledge-based approach for interpreting genome-wide expression profiles. *Proc Natl Acad Sci U S A*, *102*(43), 15545-15550. https://doi.org/10.1073/pnas.0506580102

Takasato, M., Er, P. X., Chiu, H. S., & Little, M. H. (2016). Generation of kidney organoids from human pluripotent stem cells. *Nat Protoc*, *11*(9), 1681-1692. https://doi.org/10.1038/nprot.2016.098

van den Berg, C. W., Ritsma, L., Avramut, M. C., Wiersma, L. E., van den Berg, B. M., Leuning, D. G., Lievers, E., Koning, M., Vanslambrouck, J. M., Koster, A. J., Howden, S. E., Takasato, M., Little, M. H., & Rabelink, T. J. (2018). Renal Subcapsular Transplantation of PSC-Derived Kidney Organoids Induces Neo-vasculogenesis and Significant Glomerular and Tubular Maturation In Vivo. *Stem Cell Reports*, *10*(3), 751-765. https://doi.org/10.1016/j.stemcr.2018.01.041

Wishart, D. S., Feunang, Y. D., Marcu, A., Guo, A. C., Liang, K., Vázquez-Fresno, R., Sajed, T., Johnson, D., Li, C., Karu, N., Sayeeda, Z., Lo, E., Assempour, N., Berjanskii, M., Singhal, S., Arndt, D., Liang, Y., Badran, H., Grant, J., . . . Scalbert, A. (2018). HMDB 4.0: the human metabolome database for 2018. *Nucleic Acids Res*, *46*(D1), D608-d617. https://doi.org/10.1093/nar/gkx1089

Yu, G., Wang, L. G., Han, Y., & He, Q. Y. (2012). clusterProfiler: an R package for comparing biological themes among gene clusters. *Omics*, *16*(5), 284-287. https://doi.org/10.1089/omi.2011.0118
